# Can Diet Quality Be Associated with Disease Activity in a Prospective Dutch Inflammatory Bowel Disease Cohort?

**DOI:** 10.3390/nu17081298

**Published:** 2025-04-08

**Authors:** Lola J. M. Koppelman, Corien L. Stevens, Iris Barth, Rutger J. Jacobs, Gerard Dijkstra, Andrea E. van der Meulen-de Jong, Marjo J. E. Campmans-Kuijpers

**Affiliations:** 1 Department of Gastroenterology and Hepatology, Leiden University Medical Centre, Albinusdreef 2, 2333 ZG Leiden, The Netherlands; ae.meulen@lumc.nl; 2 Department of Gastroenterology and Hepatology, University Medical Centre Groningen, University of Groningen, Hanzeplein 1, 9713 GZ Groningen, The Netherlands; i.barth@umcg.nl (I.B.); gerard.dijkstra@umcg.nl (G.D.); m.j.e.campmans-kuijpers@umcg.nl (M.J.E.C.-K.); 3 Department of Gastroenterology and Hepatology, Alrijne Hospital, Houtlaan 55, 2334 CK Leiden, The Netherlands; rjjacobs@alrijne.nl

**Keywords:** inflammatory bowel disease [IBD], diet quality, prospective cohort study

## Abstract

**Background/Objectives**: Inflammatory bowel disease (IBD) is characterized by a relapsing-remitting disease course, influenced by dietary factors. This study aims to examine diet quality in IBD patients and investigate its association with disease activity. **Methods:** In total, 477 participants from a prospective IBD cohort study at two Dutch hospitals were approached to complete a population-specific food frequency questionnaire (GINQ-FFQ) at baseline and after one year. Disease characteristics were assessed at multiple time points. Food-related Quality of Life (FrQoL) was assessed at baseline. Diet quality was measured via the Dietary Inflammatory Index (DII), Mediterranean Diet Score (MDS), Healthy Diet Indicator score (HDI), ultra-processed food intake, and Principal Component Analysis (PCA) to identify dietary patterns. The outcomes were compared to the general Dutch population. **Results**: In total, 191 participants completed the GINQ-FFQ at baseline, of whom 53 had active disease. Patients in remission had higher FrQoL than patients with active disease (*p* = 0.020). Diet quality and adherence to specific dietary patterns were not associated with disease activity. However, logistic regression showed a trend toward increased odds of increase in disease activity with an animal protein-rich pattern (OR: 1.479, *p* = 0.088) and a potential association between the Convenience diet and decreased disease activity (OR: 1.396, *p* = 0.060). Both the Dutch population and the patient cohort scored poor on all diet quality scores. **Conclusions**: The current study shows no conclusive evidence of an association between disease activity and both diet quality and dietary patterns in patients with IBD. However, the findings do suggest a possible association between animal protein-rich diets with more disease activity and Convenience-like diets with less disease activity. Furthermore, a similar diet quality was observed in IBD patients and the general Dutch population. Nevertheless, diet quality was generally poor and can be improved.

## 1. Introduction

Inflammatory bowel disease (IBD) is a complex condition marked by chronic intestinal inflammation, primarily manifesting as Crohn’s disease (CD) and ulcerative colitis (UC). In UC the disease is limited to the colon. In CD, the disease can involve the whole gastrointestinal tract, but it most commonly affects the ileum and colon. In general, patients have a relapsing–remitting course of the disease, but the disease is heterogeneous at the genetic, microbial, immunological, and clinical levels [1]. Previous studies showed that nutrition plays an important role in the development and behavior of the disease [2,3]. Certain dietary patterns are associated with increased susceptibility for inflammation and disease activity (e.g., Western diet, ultra-processed diet), whereas others (e.g., Mediterranean diet) are thought to prevent flare development [4,5,6]. Exact underlying mechanisms are not yet fully understood, but the main hypothesis revolves around the effect of nutritional compounds on the composition of the gut microbiome [7,8].

Diet quality can be assessed by several different methods. Most used are dietary quality scores, such as the dietary inflammatory index score (DII), Mediterranean diet score (MDS) and healthy diet indicator (HDI) [9,10,11]. These a priori scores, based on a predefined scoring system, evaluate adherence to a predefined dietary pattern. The DII is a diet quality score developed to assess the inflammatory potential of diets [10]. The goal of the HDI is to assess the adherence of individuals to the nutritional guidelines developed by the World Health Organization (WHO) [9]. Lastly, the MDS is a score that can assess adherence to the traditional Mediterranean diet, which has previously been linked to multiple health benefits [11,12,13]. On the other hand, a posteriori methods, such as principal component analysis (PCA), are data-driven techniques used to identify dietary patterns that are specific to a specific dataset [14]. Both methods can be used to evaluate diet quality and associations with disease outcomes.

The aim of this current study is to prospectively evaluate diet quality, both a priori and a posteriori, and ultra-processed food intake in a Dutch IBD population, and to assess whether diet quality can be associated with disease activity. A secondary aim is to investigate the changes in diet after one year of follow-up.

## 2. Materials and Methods

### 2.1. Study Population

**Figure 1:**
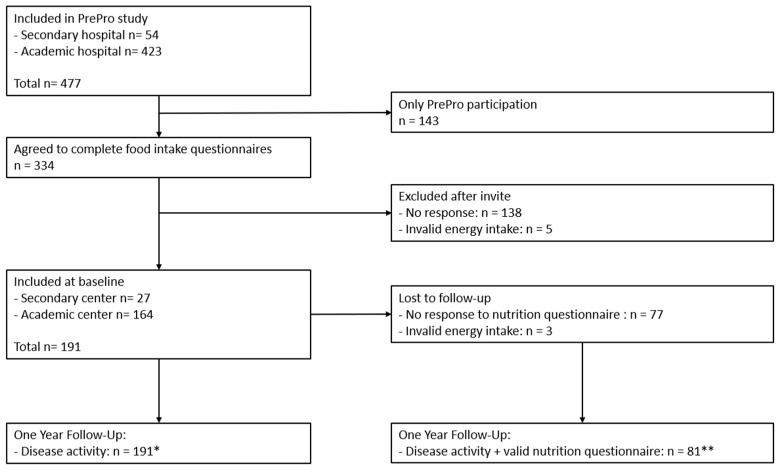
Inclusion flow-chart. PrePro study: “Prediction of IBD Disease Activity in Individual Patients Based on PROMs and Clinical Data” (NCT05578768). * = included in multinomial logistic regression analysis. ** = included in all follow-up dietary analyses.

The participants of the current study were included in a larger prospective trial called PrePro (“*Prediction of IBD Disease Activity in Individual Patients Based on PROMs and Clinical Data*” (NCT05578768)), conducted at Leiden University Medical Centre and Alrijne Hospital in The Netherlands (Figure 1). In this study, adult IBD patients visiting the outpatient clinic at one of both centers were followed over time. At the start of the PrePro study, all participants were invited to complete online questionnaires regarding food intake, food-related quality of life (FrQoL) and physical activity level (PAL). Other baseline and disease characteristics were collected from electronic patient files. Patients were recruited between October 2022 and October 2023 with a one-year follow-up period. The patients were categorized in groups regarding biochemical disease activity (definition: active disease defined as C-Reactive Protein (CRP) ≥ 5 mg/L and/or Fecal Calprotectin (FCP) ≥ 150 µg/g) and IBD phenotype (CD versus UC).

### 2.2. Diet

#### 2.2.1. Food-Related Quality of Life

Food-related quality of life was assessed using the Fr-QoL-29 questionnaire, which consists of 29 items rated on a 5-point Likert scale [15]. Total scores range from 29 to 145, with higher scores indicating better food-related quality of life. This questionnaire was administered at baseline.

#### 2.2.2. Dietary Data Collection

Dietary data were collected using the validated GINQ-FFQ (see Supplementary File S1) [16,17]. The GINQ-FFQ is a food frequency questionnaire designed for the IBD population. It consists of 121 questions to obtain information on the consumption of 218 food items and 26 food groups (see Supplementary Table S1). Information is linked to the Dutch food composition database (NEVO version 2019/2.0) [18] to obtain crude dietary intake of the patients. At baseline, the participants were asked to fill out the GINQ-FFQ through the REDCAP data capturing tool, hosted by the University Medical Center Groningen (UMCG) [19,20]. After 1 year of follow-up, participants who previously filled out the FFQ, were asked to do this again. Patients with an implausible energy intake (for women <500 and >3500 kilocalorie (kcal), and men <800 and >4000 kcal) [21,22] were excluded from analyses.

#### 2.2.3. Diet Quality Scores

To assess diet quality, three diet quality scores were calculated: DII, HDI and MDS. Also, the consumption of ultra-processed food (UPF) in this cohort was determined. These are all a priori scores, based on predefined scoring systems based on evidence with particular disease outcomes.

- Dietary Inflammatory Index

The DII is a literature-derived, population-based score developed to compare diverse populations based on the inflammatory potential of their diets. Individual food component intake was matched with a global mean intake representing different regions [10]. A z-score was calculated by comparing the reported intake with the global mean, divided by the standard deviation (SD). This score was then transformed into a standardized percentile and adjusted by the inflammatory effect of each food parameter. All of the food parameter-specific DII scores were then summed to create the overall DII score. Higher DII scores indicate a pro-inflammatory diet. In this study, 28 food components were included in the DII score: energy (kcal), alcohol, carbohydrates, fiber, protein, total fat, monounsaturated fat, poly unsaturated fat, omega-3, omega-6 fatty acid, trans fatty acids, saturated fat, cholesterol, beta-carotene, retinol, vitamin B1, vitamin B2, vitamin B3, vitamin B6, folate, vitamin B12, vitamin C, vitamin D, vitamin E, iron, magnesium, selenium, and zinc. Other components of the score were not available in the current dataset.

b. Healthy Diet Indicator

The HDI-2015 was developed by *Kanauchi* and *Kanauchi* [9] and is based on the adherence to the nutritional guidelines developed by the World Health Organization (WHO). This score consists of 7 components: an intake of fruits and vegetables ≥400 g/day; an intake of total fat <30% of total energy intake; an intake of saturated fatty acids <10% of total energy intake; an intake of polyunsaturated fatty acids between 6 and 11% of total energy intake; an intake of free sugar <10% of total energy intake; an intake of dietary fiber ≥25 g/day, and an intake of potassium ≥3500 mg/day. For each component consumed within the recommended quantity, 1 point was given. A score of 6–7 was deemed high adherence to WHO guidelines, a score of 4–5 was deemed moderate adherence and a score of 0–3 was considered low adherence.

To obtain energy percentages, Atwater factors were used in the following formulas: fat: ((nutrient × 9.0)/kcal_tot_) × 100; carbohydratess: ((nutrient × 4.0)/kcal_tot_) × 100; protein: ((nutrient × 4.0)/kcal_tot_) × 100; alcohol: ((nutrient× 7.0)/kcal_tot_) × 100.

c. Mediterranean Diet Score

The MDS is a diet quality score evaluating adherence to the traditional Mediterranean diet. The score consists of nine components, of which six are deemed beneficial (‘vegetables’, ‘legumes’, ‘fruits and nuts’, ‘cereal’, ‘fish’, and ‘ratio between monounsaturated and saturated fat intake’) and two detrimental (‘meat and poultry’ and ‘dairy products’). In 2003, Trichopoulou et al. published an article describing sex-specific median intake in g/day for these eight components in the mediterranean population [11]. When consuming more than the population median of beneficial components or less than the population median for detrimental components, 1 point per component was assigned. The 9th component was alcohol, which was scored based on crude intake. When males consume between 10 and 50 g alcohol/day, 1 point was assigned, and for females this was between 5 and 25 g alcohol/day. In all other cases, 0 points were scored. The MDS varies between 0 and 9. The higher the score, the more adherence to a traditional mediterranean diet [11].

d. Ultra-processed foods

The NOVA categorization was used to determine the consumption of ultra-processed food (UPF) in this cohort [23,24]. Food items were divided into two categories: UPF and non-UPF. When classification was unclear, the authors discussed the item until a consensus was reached. Of the 218 food items identified from the GINQ-FFQ, 100 were classified as UPFs, while the remaining 118 were categorized as either minimally processed foods, processed culinary ingredients, or processed foods. The energy percentage (EN%) from UPF per person was calculated to quantify the relative contribution of UPF to the total caloric intake, providing a clear metric for evaluating the extent of UPF consumption within the cohort. This was calculated by summing the kcal from UPF, dividing this by the total kcal consumed, and multiplying it by 100.

#### 2.2.4. General Dutch Population Reference Cohort

Data from the Dutch national food consumption survey has been used as a reference for the intake of the general Dutch population (NEVO version 2021) [18]. In this national study, two non-consecutive 24 h recall interviews were performed with 3570 children and adults between 2019 and 2021 [25]. Children <18 years and participants with implausible energy intake (for women <500 and >3500 kcal, and men <800 and >4000 kcal) [21,22] were excluded from comparison with the current IBD study cohort.

### 2.3. Statistics

#### 2.3.1. Descriptive Statistics

Baseline characteristics and dietary intake are presented as mean ± SD for normally distributed data and as median with interquartile range (IQR) for skewed data. Categorical variables are presented as absolute numbers with percentages. In the full cohort (n = 191), differences between patients in remission and those with active disease were assessed using the *t*-test for normally distributed continuous variables and the Mann-Whitney U test for skewed data. Categorical variables were compared using the Chi-square (Χ^2^) test. Furthermore, the paired samples *t*-test and the Wilcoxon signed-rank test were used to evaluate within-participant changes over time in the sub-cohort of participants who filled out the GINQ-FFQ both at baseline and at 12 months follow-up (n = 81).

A *p*-value of <0.05 was considered statistically significant.

#### 2.3.2. Dietary Pattern Analysis

In the present study, PCA was used as an a posteriori, also known as data-driven, analysis of dietary intake. PCA is a dimension reduction technique that identifies patterns, or components, based on correlations [26]. For this study, the method was used to identify dietary patterns based on correlations between our 26 food groups. The PCA was conducted with varimax rotation using a maximum of 50 iterations. Assumptions were checked using a correlation matrix, Bartlett’s Test of Sphericity, and the Kaiser–Meyer–Olkin test. Coefficients with values above 0.3 or below −0.3 were considered relevant for interpretability. The number of patterns to retain was determined by the scree plot, eigenvalues, and interpretability of the components. For each patient, a component score for each dietary pattern was calculated, using SPSS version 28.0, as a weighted sum of standardized food group intakes, with factor loadings as weights. These scores indicate how closely a patient’s diet aligns with each pattern. Per dietary pattern, Mann–Whitney U testing was performed to check for differences in these component scores, and thus adherence to the pattern, between patients with active disease and remission. This was done for the complete baseline cohort, and for the part of the cohort that completed the GINQ-FFQ both at baseline and after one year follow-up. A multinominal logistic regression was performed on all 191 patients with IBD to investigate the association between baseline adherence to dietary patterns PC1, 2, and 3 and changes in disease activity after one year of follow-up. The dependent variable was change in disease activity associated with three categories: “No Change” (reference category), “Increase”, and “Decrease”. Principal components representing dietary patterns were included as independent variables. The model estimates the odds of an increase or decrease in disease activity relative to no change.

Analyses were performed using IBM SPSS version 28.0 and RStudio (R version. 4.4.2) [27,28].

## 3. Results

### 3.1. Patient Characteristics

The study population comprised 191 patients, including 103 patients with CD and 88 with UC. Of these, 81 patients completed the GINQ-FFQ both at baseline and after one year. Baseline characteristics are shown in Table 1. At baseline, 138 patients were in biochemical remission, while 53 had active disease (active disease is defined as CRP ≥ 5 mg/L and/or FCP ≥ 150 µg/g). The median age was 50 years (IQR: 38.0–62.0), with a median disease duration of 17 years (IQR: 8.0–27.0). More disease-specific characteristics are shown in Supplementary Table S2.

Patients in remission experienced a higher food-related quality of life than those with active disease, with scores of 110 (IQR: 87–125) compared to 90 (IQR: 82–119), respectively (*p* = 0.020).

The general Dutch reference cohort of 1729 participants had a mean age of 55-years (SD ± 15-years), 49.9% was female, mean BMI was 26.7kg/m^2^ (SD ± 5.1), 38.3% had a high education level, and 25.4% had a low education level. Lastly, 75.4% of participants had a normal to active lifestyle.

### 3.2. Crude Dietary Intake

#### 3.2.1. Habitual Diet

Habitual dietary intake of macro- and micronutrients at baseline (n = 191) is shown in Table 2. There were no significant differences in nutrient intake based on disease activity. Regarding the food group levels, patients in remission consumed significantly more products within the pastries and cookies (*p* = 0.017), sugar, confectionery, sweet sauces and sweet spreads (*p* = 0.004), and herbs and spices (*p* = 0.034) groups, shown in Table 3.

#### 3.2.2. Follow-Up Data

Of 81 patients with dietary assessment data at both baseline and after 1 year follow-up, 59 patients were in remission at baseline, 55.6% were female, the mean age was 52.2 years (SD ± 1.7 years), 58.0% were patients with CD, and the median disease duration was 19 years (IQR 8.5–29.0 years). There were more patients with perianal disease in the remission group compared to patients with active disease (41.9% and 12.5%, respectively, *p* = 0.040), shown in Supplementary Table S3. After 1 year, 9 of 59 patients in remission at baseline had active disease, and of 22 patients with active disease at baseline, 9 patients were in remission. Because of the small sample size, we were just interested in differences over time in the total group of 81 patients.

Differences between nutrient and food group intake at baseline and after 12 months were tested using Wilcoxon rank or paired *t*-tests, depending on the distribution of the data. At 12 months, patients consumed significantly more total fat (E%, *p* = 0.045), mono- (*p* = 0.008) and polyunsaturated fatty acids (*p* = 0.024), potassium (*p* = 0.014), vitamin E (*p* = 0.041), and vitamin B1 (*p* = 0.012), shown in Supplementary Table S4.

After 12 months, patients also consumed more products in the fats and oils (*p* = 0.001) group and fewer products in the cheese (*p* = 0.031) group, shown in Supplementary Table S5.

### 3.3. Dietary Quality Scores and Disease Activity

Overall patients scored low on both HDI-2015 and MDS at baseline (Table 4). Patients with UC scored significantly higher on the MDS compared to patients with CD (*p* = 0.003). The mean DII score was 0.102 (SD ± 2.491), indicating that the overall diet of patients was neither very pro- nor anti-inflammatory. There were no differences in diet quality scores based on disease activity. Additionally, there were no observed differences in the consumption of ultra-processed foods between patients in remission and those with active disease. When comparing the current IBD study population to the general Dutch population, the patient population scored higher on the DII and thus followed a more inflammatory diet compared to the overall Dutch population (mean 0.102 ± 2.491 and −0.879 ± 2.512, respectively). The patient population scored better on the HDI-2015 and MDS. Consumption of UPFs was comparable between both populations. The full results are shown in Table 4.

Over one year, UC patients’ dietary quality scores indicated a significant increase in inflammatory potential, rising from 0.187 ± 2.021 at baseline to 1.072 ± 2.287 at the one-year mark (*p* = 0.036) (see Supplementary Table S6). There were no other statistical differences in dietary quality scores or UPF intake between both timepoints (Table S6).

### 3.4. Dietary Patterns and Disease Activity

To assess dietary patterns, PCA with varimax rotation was performed on food group data (1) for the complete cohort (n = 191) and (2) for the baseline of the follow-up cohort (n = 81) and (3) after 1 year of the follow-up cohort (n = 81). Based on scree plots and eigenvalues for all three groups, three dietary patterns could be distinguished, explaining about 30% of the variance in the food group data. The results are shown in Table 5.

Within the complete cohort, three dietary patterns were identified: an animal protein-rich dietary pattern (PC1), a Mediterranean-like dietary pattern (PC2), and a Convenience dietary pattern (PC3). In the follow-up cohort, dietary patterns were identified both at baseline and at the follow-up point. At baseline, a Mediterranean-like dietary pattern (PC4), a traditional Dutch dietary pattern (PC5), and a Dutch healthy dietary pattern (PC6) were identified. At the 12-month follow-up, a Mediterranean-like/Pescetarian dietary pattern (PC7), a Vegetarian dietary pattern (PC8), and a Convenience dietary pattern (PC9) were identified.

Differences in component scores, which reflect individual adherence to a dietary pattern, between patients in remission and patients with active disease were tested using Mann-Witney U or *t*-tests and are shown in Table 6. There were no statistically significant differences in adherence to a certain dietary pattern based on disease activity.

Table 7 shows the results of the multinomial logistic regression. Adherence to an animal protein-rich dietary pattern (PC1) showed a trend toward increased odds of an increase in disease activity compared to no change (Odds Ratio (OR): 1.479, 95% Confidence Interval (CI): 0.943–2.319, p = 0.088). Similarly, adherence to a Convenience diet (PC3) indicated a potential association with decreased disease activity (OR: 1.396, 95% CI: 0.986–1.976, p = 0.060). However, neither association reached statistical significance.

## 4. Discussion

Diet quality was not associated with differences in biochemical disease activity. However, an animal protein-rich dietary pattern seems to be associated with increased disease activity, whereas a Convenience dietary pattern was associated with a decrease in disease activity. However, these associations did not reach statistical significance. While baseline dietary intake showed few differences, patients in remission consumed more pastries, cookies, and sugary products. After one year, notable dietary changes included increased intake of mono- and polyunsaturated fats, potassium, vitamin B1, and vitamin E, alongside shifts in food group consumption, such as increased dairy and fats, but reduced cheese and non-alcoholic beverages, suggesting a shift toward healthier fats. UC patients showed a significant increase in dietary inflammatory potential after one year.

### 4.1. Diet Quality

This cohort scored low on all diet quality measures. Poor adherence to healthy eating guidelines, as reflected in low HDI-2015 and MDS scores, has been linked to chronic low-grade inflammation, while higher adherence is associated with lower CRP and interleukin levels [13,29,30,31,32,33]. Despite these low scores, this cohort performed slightly better on the HDI-2015 and MDS compared to the general Dutch population.

A pro-inflammatory diet, measured by the DII, is linked to increased disease activity in IBD patients [34,35]. In the current cohort, patients with UC had a significant increase in DII score after 1-year follow-up. When studying changes in food group and nutrient intake, these can correlate with a decreased consumption of fiber. However, since disease activity remained relatively stable over time and follow-up data on FrQoL was lacking, and diet was not routinely supervised during the study period, there is no clear explanation for this change in intake and DII score. Given these constraints, this finding should be interpreted cautiously. Despite scoring relatively better on the MDS and HDI-2015 scores, the DII score shows that the current patient cohort seems to follow a more pro-inflammatory diet compared to the general Dutch population.

These results highlight room for improvement in diet quality and underscore the need for proactive dietary support and greater involvement of dietitians in routine care.

### 4.2. Disease Activity and Dietary Habits

No clear differences in habitual intake or diet quality were observed based on disease activity, suggesting that dietary patterns remain stable despite fluctuations in disease state. A possible explanation is the high FrQoL in the current cohort. Huges et al. describes mean FrQoL scores of 89.5 (SD 28.6), whereas Guadagnoli et al. describes mean scores of 91.2 (SD 26.5) in patients in remission and mean scores of 67.7 (SD 19.6) in patients with active disease [15,36]. The current cohort scored evidently higher, especially in the patient group with active disease. Most patients included were treated in an academic hospital where the IBD doctors and nurses spend a lot of time on nutritional education at the outpatient IBD clinic. Also, a dietician is easily accessible. Although this cannot be quantified, this might play a role in the outcome of FrQOL. The patients were not informed of their scores on FrQoL. However, when they perceived a good FrQoL, they were probably less inclined to make changes in their dietary habits to control symptoms related to disease [37,38].

Another explanation for similar intake patterns, independent of disease activity, could be the relatively long median disease duration of 17 years (IQR [8.0–27.0] in the current cohort. It is known that dietary habits can be influenced by environmental, social, and individual factors [39]. However, when those factors are stable, dietary habits also remain stable. Disease and changes in disease status are individual factors. Patients most often change their dietary habits when disease is most severe [40,41]. This most often is at times of new diagnosis and when searching for the best treatment strategy. This can also be an explanation for patients in remission consuming more pastries, cookies, and sugary products. These patients have a stable, remitted disease and most likely no incentive to change their dietary habits to improve disease outcomes. Previously, Peters et al. described how patients with IBD consume more sugar and sweets when compared to controls [42].

A trend was observed where a Convenience diet may have been associated with reduced disease activity. This stands in contrast with studies describing ultra-processed foods and diets negatively impacting IBD [43,44]. A possible explanation for this finding aligns with the above-described incentive needed to change dietary behaviors. Furthermore, Zallot et al. and de Vries et al. previously described how patients are more inclined to exclude foods they know to be detrimental to cope with disease symptoms [40,41]. Overall, symptoms are more prevalent and debilitating during periods of active disease. Knowing this, we can hypothesize that when patients are in remission and experience fewer or no symptoms, they feel freer to consume all foods.

### 4.3. Dietary Advice for Patients with IBD

Current European guidelines lack any description of the use of specific diets or dietary patterns to maintain remission in IBD, even though evidence is accumulating to suggest that specific diets and dietary components can be pro- or anti-inflammatory [12,13,45]. The European Society for Clinical Nutrition and Metabolism (ESPEN) published a practical guideline on clinical nutrition in inflammatory bowel disease in 2023 [46]. This guideline is extensive, with 71 recommendations in total, ranging from recommendations regarding the prevention of IBD to recommendations regarding, for example, the use of pre- and/or probiotics, and recommendations for the remission phase and peri-operative phase. However, many of the recommendations are based on good clinical practice, and thus expert opinions. Furthermore, real nutritional recommendations for patients with UC are lacking. For CD, the CD Exclusion Diet is considered for remission induction therapy. However, this is based on evidence with a high probability of non-causality. There are no recommendations about how diet can support maintenance of remission, other than following a healthy dietary pattern [46]. The European Crohn’s and Colitis Organisation (ECCO) guidelines only recommend the use of exclusive enteral nutrition (EEN) for remission induction in a very specific group of patients with CD [44]. Other recommendations are centered around optimizing nutritional status to improve surgical outcomes [47,48,49,50].

When comparing the dietary habits of the current cohort to the recommendations made by ESPEN and ECCO, it can be observed that both patients in remission and with active disease do not follow the recommended “healthy dietary pattern” as described by the Dutch Institute for Public Health and Environment (RIVM) [51]. Participants do not eat enough fruits, vegetables, legumes, nuts, and fish. Furthermore, as described in the ESPEN guidelines, it needs to be acknowledged that these patients have a chronic illness, and thus the general advice described for a healthy population is not always appropriate for patients with IBD [46].

Observing the data from the general Dutch population cohort reveals that adhering to a healthy diet, as described by the RIVM, proves to be difficult, in general, and is not specific to IBD patients [51]. This can be due to a lack of knowledge of what is considered healthy, but also a lack of personal motivation to follow recommendations.

### 4.4. Strengths and Limitations

A key strength of this study is its comprehensive assessment of dietary intake in IBD patients, providing insights into multiple dietary facets, including nutrient intake, food group consumption, dietary patterns, and diet quality. By incorporating both a priori and a posteriori methods to evaluate dietary data, this study combines predefined diet quality scores with data-driven dietary pattern analysis, allowing for a more nuanced understanding of diet in this IBD cohort. This dual approach enhances the robustness of dietary assessment.

A limitation of this study is the relatively small number of participants who completed the one-year follow-up questionnaire, which may limit the generalizability of longitudinal findings. Moreover, the relatively small sample size may have limited the ability to perform subgroup analyses, and the observed trends in the regression analysis should be interpreted with caution due to risk of type-2 error. In addition, disease activity can be influenced by various other factors such as medication, therapies, stress, and environmental factors, which may complicate the interpretation of the observed relationship between diet and disease activity. Regarding the comparison of this cohort with the general Dutch population, it should be noted that the data for the general population were collected using two 24 h recalls. Unlike a FFQ, which captures long-term dietary patterns, 24 h recalls rely on short-term memory and may not fully represent habitual intake, especially for infrequently consumed foods. Since 24 h recalls and FFQ’s are very different ways of evaluating dietary intake, a direct comparison between data derived from these two different methods should be performed with caution. Moreover, although the use of the GINQ-FFQ and comparison to the Dutch general population provides valuable local context, this limits generalizability to other populations. Dutch dietary patterns—such as high dairy and bread consumption—may differ from those in regions like the US or Asia. While some findings may reflect general nutritional principles (e.g., fiber intake or ultra-processed food consumption), others likely reflect local habits.

Lastly, both this cohort and the Dutch population score low on the MDS, which may partly reflect its limitations in a Western context. However, it is still of interest to test the Western population on the original Mediterranean diet using the MDS as described by Trichopoulou et al. [11], since its anti-inflammatory effects have been broadly investigated and proven.

### 4.5. Implications

Both patients and healthcare providers are in need of more specific recommendations, based on stronger types of evidence. Intervention studies that can support this kind of evidence are currently ongoing [52,53,54]. These studies can also provide incentive and tools for healthcare providers to incorporate dietary care into standard IBD care. Descriptive studies, like the current study, are necessary to provide deeper insight into dietary habits of the IBD patient population. This again can be the basis for the population-specific design of dietary interventions.

## 5. Conclusions

The current study did not find conclusive evidence for an association between diet quality and disease activity. However, diet quality in Dutch patients with IBD was poor overall, highlighting room for improvement and stressing the importance of paying more attention to patients’ dietary habits for healthcare providers. In addition, our findings do suggest the possibility that protein-rich diets may be associated with more disease activity and that Convenience-like diets may be associated with less disease activity.

## Figures and Tables

**Table 1:** Baseline characteristics.

		Disease Activity		
	IBD Total n = 191	Remission n = 138	Active Disease ^a^ n = 53	*p*-Value
Age (years)	50.0 [38.0–62.0]	51.0 [39.0–63.0]	43.0 [37.0–58.0]	0.208
Female sex (n, %)	103 (53.9)	75 (54.3)	28 (52.8)	0.851
Education level				0.678
Low	12 (6.7)	10 (7.7)	2 (4.1)	
Intermediate	73 (40.8)	53 (40.8)	20 (40.8)	
High	94 (52.5)	67 (51.5)	27 (55.1)	
BMI (kg/m^2^)	24.4 [21.9–27.6]	24.6 [22.2–28.1]	23.7 [21.2–25.7]	0.079
PAL				0.575
Minimally active	40 (20.9)	26 (18.8)	14 (26.4)	
Moderately active	100 (54.4)	73 (52.9)	27 (50.9)	
Moderately active with intensive sport	38 (19.9)	30 (21.7)	8 (15.1)	
Highly active	13 (6.8)	9 (6.5)	4 (7.5)	
Disease duration (years)	17.0 [8.0–27.0]	17.0 [9.0–28.0]	15.0 [8.0–24.0]	0.238
IBD type (CD) (n, %)	103 (53.9)	74 (53.6)	29 (54.7)	0.892
C-reactive protein (mmol/L) ^b^	2.15 [1.00–5.30]	2.00 [1.00–3.65]	4.40 [1.00–9.20]	0.029
Fecal calprotectin (µg/g) ^c^	60.00 [16.00–233.50]	24.00 [12.00–94.00]	449.50 [192.00–1148.50]	<0.001 *
FrQoL	104 [85–124]	110 [87–125]	90 [82–119]	0.020 *

Data are presented as median [interquartile ranges] or as absolute numbers of available data (=n) with corresponding percentages (%). Mann–Whitney U test or Chi-square (Χ^2^) test was used to compare between active disease and remission. Education level is defined as follows: Low includes primary education and secondary education. Intermediate refers to vocational education. High includes higher professional education and academic education. BMI: Body Mass Index, PAL: physical activity level, IBD: inflammatory bowel disease, CD: Crohn’s disease, FrQoL: Food-related Quality of Life score, GI: gastrointestinal tract. ^a^: defined as CRP ≥ 5 mg/L and/or FCP ≥ 150 µg/g. ^b^: n = 130. ^c^: n = 100. * *p*-value < 0.05.

**Table 2:** Habitual diet of the complete baseline group (n = 191).

			Disease Activity		
	General Dutch Population ^a^	IBD Total n = 191	Remission n = 138	Active Disease ^b^ n = 53	*p*-Value
Macronutrients					
Energy total (kcal)	2039.44 ± 565.75	2066.52 ± 610.05	2101.24 ± 602.16	1976.14 ± 626.89	0.205
Protein					
-Total (g)	79.89 ± 23.27	84.83 ± 30.45	85.61 ± 30.91	82.81 ± 29.43	0.057
-Plant (g)	29.72 [23.91–37.33]	27.73 [20.82–35.14]	27.87 [20.92–35.24]	26.82 [19.62–34.39]	0.630
-Animal (g)	48.6 ± 20.14	55.58 ± 25.58	56.12 ± 26.81	54.18 ± 22.24	0.641
Carbohydrates					
-Total (g)	207.44 ± 68.84	193.78 ± 72.79	196.68 ± 70.75	186.23 ± 78.04	0.376
-Sugar (g)	93.06 ± 33.05	87.86 ± 36.32	89.41 ± 34.99	83.82 ± 39.65	0.343
-Fiber (g)	20.27 [16.14–25.47]	22.32 [16.83–29.97]	21.57 [16.92–30.03]	23.78 [16.41–29.58]	0.852
Alcohol (g)	0.00 [0.00–12.9]	1.48 [0.00–5.71]	1.78 [0.00–6.48]	0.89 [0.00–3.29]	0.070
Water (g)	2930.66 ± 833.2	1945.73 ± 645.76	1930.16 ± 625.23	1986.28 ± 701.05	0.592
Fat					
-Total (g)	86.7 ± 30.47	96.48 ± 31.62	98.30 ± 31.34	91.75 ± 32.16	0.201
-Total (E%)	38.03 ± 7.10	42.33 ± 7.48	42.43 ± 7.33	42.09 ± 7.93	0.785
-Saturated fat (g)	29.56 [22.69–37.90]	27.61 [21.79–35.86]	28.66 [23.00–36.38]	26.58 [20.01–32.43]	0.143
-MUFA (g)	29.7 [22.62–38.66]	37.99 [31.28–47.11]	38.08 [31.21–49.69]	37.35 [32.27–43.60]	0.428
-PUFA (g)	16.41 ± 7.54	19.04 ± 7.57	19.57 ± 7.46	17.66 ± 7.75	0.119
-PUFA N3 (g)	11.72 [8.44–16.16]	2.81 [2.14–4.07]	2.88 [2.24–4.16]	2.75 [2.06–3.72]	0.401
-PUFA N6 (g)	1.77 [1.26–2.45]	13.63 [10.47–16.89]	13.85 [10.96–17.59]	13 [9.79–15.88]	0.100
-Trans fatty acids (g)	0.61 [0.4–0.89]	0.62 [0.42–0.83]	0.66 [0.42–0.86]	0.55 [0.38–0.72]	0.059
-Cholesterol (g)	195.5 [135–287.59]	222.02 [167.73–297.22]	216.31 [166.76–288.89]	227.83 [171.69–321.63]	0.563
Micronutrients					
Sodium (mg)	2289.18 ± 807.04	1890.64 ± 763.96	1932.45 ± 770.89	1781.78 ± 741.70	0.223
Potassium (mg)	3285.66 ± 933.78	3159 ± 940.03	3190.50 ± 900.44	3078.31 ± 1040.85	0.462
Calcium (mg)	1016.17 ± 401.08	948.04 ± 430.40	972.51 ± 419.43	884.22 ± 455.64	0.205
Magnesium (mg)	347.99 ± 104.41	337.48 ± 110.71	340.11 ± 106.68	330.62 ± 121.40	0.597
Iron					
-Total (mg)	10.45 ± 3.15	10.14 ± 3.46	10.25 ± 3.58	9.85 ± 3.12	0.471
-Haeme (mg)	0.56 [0.06–1.29]	0.90 [0.51–1.42]	0.90 [0.40–1.52]	0.85 [0.63–1.33]	0.800
-Non-haeme (mg)	9.48 ± 2.98	9.02 ± 3.10	9.10 ± 3.18	8.80 ± 2.92	0.554
-Zinc (mg)	10 [8.02–12.29]	9.86 [8.20–12.44]	9.89 [8.47–12.44]	9.79 [7.57–11.40]	0.492
Vitamins					
A (RAE) (mug)	624.5 [440.89–933.74]	787.79 [609.46–1059.07]	823.73 [648.17–1074.05]	713.84 [589.36–979.46]	0.094
D (mug)	3.06 ± 2.39	3.71 ± 1.98	3.74 ± 2.08	3.61 ± 1.70	0.693
E (mg)	11.83 [8.27–16.66]	13.91 [10.66–18.09]	14.21 [10.99–18.10]	12.85 [10.37–16.50]	0.089
B1 (mg)	1.05 ± 0.43	0.98 ± 0.38	0.99 ± 0.38	0.94 ± 0.37	0.398
B2 (mg)	1.43 ± 0.55	1.31 ± 0.56	1.35 ± 0.56	1.22 ± 0.56	0.161
B3 (mg)	17.8 ± 7.21	21.94 ± 8.88	21.93 ± 8.76	21.96 ± 9.25	0.984
B6 (mg)	1.56 ± 0.55	1.69 ± 0.57	1.68 ± 0.57	1.70 ± 0.59	0.798
B11 (mug)	280.9 ± 108.56	249.85 ±77.33	252.00 ± 76.83	244.23 ± 79.09	0.536
B12 (mug)	4.47 ± 3.57	4.83 [3.23–6.81]	4.81 [3.23–6.78]	4.86 [3.33–6.88]	0.958
C (mg)	94.03 ± 58.86	91.34 ± 47.18	90.55 ± 44.49	93.39 ± 55.98	0.710

Values are shown as median [IQR] or mean ± SD. IQR = interquartile range, SD = standard deviation. IBD = inflammatory bowel disease. ^a^ Derived from the Dutch National Food Consumption Survey, n = 1729. ^b^: defined as CRP ≥ 5 mg/L and/or FCP ≥ 150 µg/g. Mann-Witney U or *t*-test was performed to assess difference between active disease and remission. n = number. E% = Energy percent = ((nutrient × Atwater factor)/Energy total) × 100. MUFA = Monounsaturated fatty acids. PUFA = Polyunsaturated fatty acids. RAE = retinol equivalents.

**Table 3:** Habitual intake of 26 food groups in 191 IBD patients.

			Disease Activity		
	General Dutch Population ^a^	IBD Total n = 191	Remission n = 138	Active Disease ^b^ n = 53	*p*-Value
Dairy and dairy products (g)	249.15 [105.84–429.09]	185.39 [84.33–377.44]	192.43 [91.75–387.71]	173.41 [71.64–264.69]	0.204
Cheese (g)	35.58 [15.50–67.20]	23.93 10.36–42.50]	26.07 [11.43–44.29]	21.25 [7.17–36.07]	0.191
Eggs (g)	0.00 [0.00–25.00]	14.29 [7.14–28.57]	14.29 [4.64–28.57]	14.29 [7.14–28.75]	0.114
Grain products and binding agents (g)	45.00 [0.00–108.00]	55.36 [30.05–100.00]	52.99 [27.43–95.71]	57.14 [32.65–108.96]	0.401
Bread (g)	107.50 [70.00–152.50]	84.87 [32.14–134.76]	88.83 [32.46–134.76]	76.67 [28.61–130.95]	0.604
Pastries and cookies (g)	28.50 [0.00–60.50]	18.39 [6.61–33.3]	20.13 [7.32–36.96]	12.95 [4.11–29.02]	0.017 *
Savory sandwich spread (g)	0.00 [0.00–0.00]	1.07 [0.00–4.82]	00.00 [0.00–4.29]	1.61 [0.00–5.36]	0.476
Savory sauces (g)	19.68 [6–41.46]	10.00 [4.64–20.45]	9.87 [4.91–18.93]	10.36 [3.75–21.43]	0.886
Savory snacks (g)	0.00 [0.00–20.00]	10.36 [3.39–20.00]	10.09 [4.29–20.00]	11.43 [2.86–20.00]	0.815
Sugar, confectionery, sweet sauces, and sweet spreads (g)	15.08 [4–33.29]	15.14 [6.61–27.00]	16.91 [7.14–29.57]	11.27 [4.45–18.61]	0.004 *
Fruit (g)	125.31 [45.12–216.95]	150.00 [75.00–300.00]	153.01 [75.00–300]	150.00 [75.00–289.29]	0.995
Nuts and seeds (g)	3.60 [0.00–25.00]	13.21 [1.43–28.75]	13.04 [0.89–30.80]	13.21 [2.68–25.00]	0.830
Vegetables (g)	149.50 [92.09–223.09]	112.78 [72.86–175.89]	114.11 [70.98–171.32]	112.14 [80.71–203.04]	0.512
Legumes (g)	0.00 [0.00–0.00]	5.36 [0.00–14.29]	5.36 [0.00–14.29]	7.14 [0.00–14.29]	0.465
Potatoes and root vegetables (g)	60.00 [0.00–113.00]	42.86 [26.79–77.68]	42.86 [26.79–78.57]	42.86 [17.86–64.29]	0.236
Ready-made meals (g)	-	21.43 [0.00–53.57]	21.43 [0.00–53.57]	21.43 [10.71–53.57]	0.911
Soups (g)	0 [0–75.07]	6.87 [2.23–17.86]	7.01 [2.23–17.86]	5.10 [1.79–17.86]	0.740
Herbs and spices (g)	3.6 [0–25]	0.29 [0.00–0.89]	0.36 [0.00–0.98]	0.11 [0.00–0.63]	0.034 *
Fats and oils (g)	19.91 [10.81–30.82]	31.51 [25.45–40.26]	31.79 [24.30–41.19]	30.81 [27.86–39.63]	0.873
Fish (g)	0 [0–15]	22.32 [8.93–46.43]	22.32 [8.93–44.64]	33.48 [22.32–49.55]	0.138
Meat and poultry (g)	60.02 [28.12–98]	100.00 [53.57–157.14]	101.79 [53.57–155.36]	92.86 [53.57–157.14]	0.982
Meat and dairy substitutes (g)	0.00 [0.00–0.00]	0.00 [0.00–0.00]	0.00 [0.00–0.00]	0.00 [0.00–0.00]	0.545
Cold cuts (g)	12.5 [0–30.94]	7.5 [0.54–18.57]	8.04 [1.07–21.43]	6.43 [0.00–16.07]	0.302
Alcoholic beverages (g)	0 [0–150]	17.85 [0.00–62.14]	22.02 [0.00–64.29]	7.14 [0.00–41.96]	0.079
Non-alcoholic beverages (g)	1942.03 ± 763.3	1090.11 ± 502.86	1064.56 ± 500.34	1156.63 ± 508.08	0.258
Miscellaneous (g)	0.00 [0.00–0.00]	0.00 [0.00–0.00]	0.00 [0.00–0.00]	0.00 [0.00–0.00]	0.380

Values are shown as median [IQR] or mean ± SD. IQR = interquartile range, SD = standard deviation. IBD = inflammatory bowel disease. ^a^ Derived from the Dutch National Food Consumption Survey, n = 1729. ^b^: defined as CRP ≥ 5 mg/L and/or FCP ≥ 150 µg/g. Mann-Witney U or *t*-test was performed to assess differences between active disease and remission. * *p*-value < 0.05. n = number.

**Table 4:** Dietary quality scores.

			Disease Activity		
	General Dutch Population ^a^	IBD Total n = 191	Remission n = 138	Active Disease ^b^ n = 53	*p*-Value
DII	−0.879 ± 2.512	0.102 ± 2.491	0.211 ± 2.384	−0.181 ± 2.755	0.332
UC		0.244 ± 2.600	0.368 ± 2.386	−0.088 ± 3.136	0.523
CD		−0.020 ± 2.399	0.074 ± 2.390	−0.259 ± 2.250	0.529
HDI-2015					0.813
Low	1661 (96.1)	160 (83.8)	115 (83.3)	45 (84.9)	
Moderate	68 (3.9)	30 (15.7)	22 (15.9)	8 (15.1)	
High	0 (0)	1 (0.5)	1 (0.7)	0 (0.0)	
UC					0.774
Low		71 (80.7)	52 (81.3)	19 (79.2)	
Moderate		16 (18.2)	11 (17.2)	5 (20.8)	
High		1 (1.1)	1 (1.6)	0 (0)	
CD					0.547
Low		89 (86.4)	63 (85.1)	26 (89.7)	
Moderate		14 (13.6)	11 (14.9)	3 (10.3)	
High		0 (0)	0 (0)	0 (0)	
MDS	2 [1–3]	3 [2–4]	3 [2–4]	3 [2–4]	0.368
UC		3 [2–4] **	3 [2–4]	3 [2–4]	0.731
CD		2 [2,3] **	2 [2,3]	3 [2–4]	0.152
UPF (EN%)	39.55 ± 13.71	42.44 ± 14.24	43.00 ± 13.57	41.00 ± 15.9	0.387
UC		40.38 ± 14.21	40.69 ± 13.98	39.58 ± 15.09	0.747
CD		44.20 ± 14.10	44.00 ± 12.97	42.17 ± 14.72	0.364

Numbers are shown as mean ± SD for DII and UPF scores, n (%) for HDI score, and median IQR score for MDS. Independent *t*-test, Mann–Whitney U test or Chi-square (Χ^2^) test was used to compare between active disease and remission. IBD = inflammatory bowel disease. UC = ulcerative colitis, n = 88. CD = Crohn’s disease, n = 103. ^a^ Derived from the Dutch National Food Consumption Survey, n = 1729. ^b^: defined as CRP ≥ 5 mg/L and/or FCP ≥ 150 µg/g. ** comparison of MSD between UC and CD: *p* = 0.003.

**Table 5:** PCA on food group data using varimax rotation.

	Complete IBD Cohort (n = 191)			Follow-Up IBD Cohort (n = 81)					
				Baseline			Follow-Up		
	PC1	PC2	PC3	PC4	PC5	PC6	PC7	PC8	PC9
Dairy and dairy products							0.404		
Cheese					0.607				
Eggs						0.629	0.682		
Grain products and binding agents			0.767			0.775	0.795		
Bread		−0.338			0.671				0.485
Pastries and cookies									
Savory sandwich spread									0.622
Savory sauces		0.784		0.396					0.690
Savory snacks								−0.646	
Sugar, confectionery, sweet sauces, and sweet spreads									
Fruit	−0.486							0.778	
Nuts and seeds	−0.597	0.353		0.606		0.335	0.348	0.644	
Vegetables		0.725		0.722			0.447	0.348	
Legumes				0.782					
Potatoes and root vegetables					0.696	0.316	0.484		
Ready-made meals			0.694						0.755
Soups				0.743					
Herbs and spices									0.312
Fats and oils					0.340				
Fish							0.357		
Meat and poultry	0.671		0.335	−0.376		0.504		−0.354	
Meat and dairy substitutes									
Cold cuts	0.594			−0.328	0.666		−0.322		
Alcoholic beverages					0.656				
Non-alcoholic beverages							0.570		
Miscellaneous									
Variance explained (%)	10.35	8.84	6.71	13.44	8.92	8.08	14.52	9.84	7.87

Only values of >0.3 (high intake, dark color) and <−0.3 (low intake, light color) are considered relevant and are shown; n = number; complete cohort (blue) = baseline food group data of all 191 patients with IBD. IBD = inflammatory bowel disease. PC1 = Animal protein-rich; PC2 = Mediterranean-like; PC3 = Convenience diet; follow-up cohort = food group data of all patients with follow-up food group data; baseline (orange): PC4 = Mediterranean-like; PC5 = traditional Dutch; PC6 = Dutch healthy diet; Follow-up (green): PC7 = Mediterranean like/Pescetarian; PC8 = vegetarian; PC9 = Convenience diet.

**Table 6:** Association between disease activity and PCA factor loadings.

		Disease Activity ^†^				
		Remission		Active Disease		
			Mean Rank		Mean Rank	*p*-Value
Complete IBD cohort (n = 191)	PC1	−0.03 ± 1.07		−0.08 ± 0.81		0.513
	PC2	−0.20 [−0.61; 0.38]	94.60	−0.16 [−0.46; 0.37]	99.64	0.573
	PC3	−0.24 [−0.61; 0.37]	92.49	0.03 [−0.37; 0.39]	105.13	0.157
Follow-up IBD cohort Baseline (n = 81)	PC4	−0.28 [−0.67; 0.35]	40.03	−0.21 [−0.66; 0.78]	43.59	0.545
	PC5	−0.19 [−0.59; 0.51]	43.54	−0.38 [−0.83; −0.02]	34.18	0.111
	PC6	−0.18 [−0.67; 0.42]	40.56	−0.11 [−0.54; 0.44]	42.18	0.782
Follow-up IBD cohort 1-year (n = 81)	PC7	−0.10 [−0.57; 0.33]	42.24	−0.24 [−0.65; 0.18]	37.68	0.438
	PC8	0.01 ±1.04		−0.02 ± 0.90		0.928
	PC9	−0.18 [−0.77; 0.56]	41.61	−0.13 [−0.68; 0.42]	39.36	0.702

Values are factor loadings. Shown as medians with [IQR] or Mean ± SD. IQR = interquartile range, SD = standard deviation. IBD = inflammatory Bowel Disease. n = number; ^†^ = disease activity at baseline. Differences in adherence to PC based on disease activity were assessed with Mann-Witney U testing or *t*-test. PC1 = Animal protein-rich; PC2 = Mediterranean-like; PC3 = Convenience diet; Follow-up cohort = food group data of all patients with follow-up food group data; Baseline: PC4 = Mediterranean-like; PC5 = Traditional Dutch; PC6 = Dutch healthy diet; Follow-up: PC7 = Mediterranean like/Pescetarian; PC8 = vegetarian; PC9 = Convenience diet.

**Table 7:** Multinomial logistic regression between baseline dietary patterns and changes in disease activity after one year.

	Change Disease Activity	B	Std. Error	OR	95% CI for OR	*p*-Value
PC1	Increase	0.391	0.230	1.479	0.943–2.319	0.088
	Decrease	−0.121	0.193	0.886	0.608–1.293	0.531
PC2	Increase	−0.029	0.242	0.972	0.604–1.562	0.906
	Decrease	−0.079	0.186	0.924	0.632–1.352	0.685
PC3	Increase	0.227	0.233	1.255	0.795–1.976	0.329
	Decrease	0.333	0.177	1.396	0.986–1.976	0.060

Multinomial logistic regression based on the total cohort (n = 191) between component scores of the three identified dietary patterns and disease activity after one year. The reference category for disease activity change is “No Change”. PC1 = Animal protein-rich; PC2 = Mediterranean-like; PC3 = Convenience diet.

## Data Availability

The data underlying this article can be shared on reasonable request to the principal investigators of the respective cohorts and in line with European directives.

## Supplementary Materials

The following supporting information can be downloaded at https://www.mdpi.com/article/10.3390/nu17081298/s1, Supplementary File S1: English translation of the GINQ-FFQ; Table S1: Food items and food groups derived from the GINQ-FFQ; Table S2. Disease-specific characteristics; Table S3. Patient characteristics of 81 participants with dietary intake data at baseline and 1-year follow-up; Table S4. Nutrient intake baseline versus follow-up (n = 81); Table S5. Food group intake baseline versus follow-up (n = 81); Table S6. Changes in dietary quality scores over the course of one year.

## Abbreviations

The following abbreviations are used in this manuscript:

CD	Crohn’s Disease
CI	Confidence Interval
CRP	C-reactive Protein
DII	Dietary Inflammatory Index
ECCO	European Crohn’s and Colitis Organisation
EEN	Exclusive Eneral Nutrition
EN%	Energy Percentage
ESPEN	European Society for Clinical Nutritition and Metabolism
FCP	Fecal Calprotectin
FFQ	Food Frequency Questionaaire
FrQoL	Food related Quality of Life
GINQ	Groningen IBD Nutritional Questionnaires
HDI	Healthy Diet Indicator
IBD	Inflammatory Bowel Disease
IQR	Inter Quartile Range
kcal	Kilo Calorie
MDS	Mediterranean Diet Score
MUFA	Monounsaturated fatty acids
OR	Odds Ratio
PAL	Physical Activity Level
PCA	Principal Component Analysis
PUFA	Polyunsaturated fatty acids
RAE	Retinol Equivalents
RIVM	Rijksinstituut voor Volksgezondheid en Milieu (Dutch Institute for Public Health and Environment)
SD	Standard Deviation
UC	Ulcerative Colitis
UPF	Ultra-Processed Food
WHO	World Health Organization
