# Supplementary material for: Can Diet Quality Be Associated with Disease Activity in a Prospective Dutch Inflammatory Bowel Disease Cohort?
Source: Nutrients. 2025 Apr 8;17(8):1298. doi: 10.3390/nu17081298 (PMC12029865; doi:10.3390/nu17081298)
Supplement: Supplementary file 1 [file nutrients-17-01298-s001.zip › nutrients-3549635-supplementary/Supplementary file 1. Groningen IBD Nutritional Questionnaires (GINQ-FFQ)_English translation.pdf]

Groningen IBD Nutritional Questionnaires - Food Frequency Questionnaire (GINQ-FFQ)

Please read the following instructions before filling out the questionnaire!

You are about to fill out a questionnaire about your dietary intake. There are a couple things to keep in mind when filling out this questionnaire:

- The questions are about the foods and beverages you have consumed in the PREVIOUS MONTH; meaning the previous 4 weeks. Including weekdays and weekend days, including birthday parties, weddings, and other non-regular days.
- For some questions, information about the way a meal is prepared is required. If you are usually not the person who prepares your meals, these questions may be difficult to answer for you. Hence, it can be of use to fill out this questionnaire together with the person who usually prepares your food.
- It is important that you fill out what YOU have eaten. It is not relevant what other family members, roommates or persons belonging to the same household have eaten.
- If you did NOT use a specific product or food item in the last month, please select: "not used".
- Sometimes you will be asked to choose between: rarely/never, sometimes, often or usually/always. Sometimes; meaning less than half of the times, often; meaning more than half of the times.
- It is NOT relevant in MOST questions WHEN a specific product or food item is consumed; thus sum the amount of product taken in over the complete day. In some questions the specific moment of consumption is relevant, then, this will be specified in the question.
- Do not take too much time to think about every question. Your first insight is often correct.
- Some questions need an additional explanation, this information is then specified in a subsentence under the main question.
- If you would like to comment on the questionnaire or specific questions, you can find a comment box at the end of the questionnaire.

I wish you succes.

*Required

Introduction questions

1. What is your participant number? *

2. In what year were you born? *

3. What are the 4 digits of your ZIP code? *

____

4. Did you adhere to a certain diet in the last month? *

Mark only one oval.

- Yes, always
- Yes, sometimes
- No

5. If yes, to what type of diet did you adhere in the last month? *

Mark only one oval.

- Not applicable
- Kilocalorie-restricted diet
- Sodium-restricted diet
- Fat-restricted diet
- Diabetes mellitus diet
- Cholesterol diet
- Fiberrich diet
- Fiber-restricted diet
- Lactose free diet
- FODMAP diet
- Gluten free diet
- Low-carbohydrate diet
- Lightly digestible diet
- Paleo diet
- Other type of diet

6. Did you adhere to a certain regimen in the last month? *

Mark only one oval.

- Yes, always
- Yes, sometimes
- No

7. If yes, to what type of regimen did you adhere in the last month? *

Mark only one oval.

- Not applicable
- Vegetarian
- Pescotarian
- Vegan
- Macrobiotic
- Anthroposophic
- Other type of regimen

8. Do you have allergies to certain foods? *

Mark only one oval.

- Yes
- No

9. What type of allergy do you have? *

Mark only one oval.

- Not applicable
- Nut allergy
- Peanut allergy
- Crustacean and shellfish allergy
- Fruit allergy
- Other: ____________________

10. What physical reaction occurs after ingestion of the specific allergen/food item?

11. Who prepares your dinner? *

Mark only one oval.

- Usually I am preparing my dinner
- Usually somebody else is preparing my dinner

12. How often have you consumed breakfast in the last month? *

Mark only one oval.

- Not used
- 1 day in 4 weeks
- 2-3 days in 4 weeks
- 1 day per week
- 2 days per week
- 3 days per week
- 4 days per week
- 5 days per week
- 6 days per week
- 7 days per week

13. How often have you consumed bread as a meal (breakfast, lunch and/or dinner) in the last month? *

Mark only one oval.

- Not used
- 1 day in 4 weeks
- 2-3 days in 4 weeks
- 1 day per week
- 2 days per week
- 3 days per week
- 4 days per week
- 5 days per week
- 6 days per week
- 7 days per week

14. How often have you consumed bread as a meal on such day? *

Mark only one oval.

- 0
- 1
- 2
- More that 2

15. How often have you consumed a warm meal (breakfast, lunch and/or dinner) in the last month? *

Mark only one oval.

- Not used
- 1 day in 4 weeks
- 2-3 days in 4 weeks
- 1 day per week
- 2 days per week
- 3 days per week
- 4 days per week
- 5 days per week
- 6 days per week
- 7 days per week

16. How often have you consumed a warm meal on such day? *

Mark only one oval.

- 0
- 1
- 2
- More that 2

Breakfast products

17. How often have you consumed breakfast drinks in the last month? *

Mark only one oval.

- Not used
- 1 day in 4 weeks
- 2-3 days in 4 weeks
- 1 day per week
- 2 days per week
- 3 days per week
- 4 days per week
- 5 days per week
- 6 days per week
- 7 days per week

18. How many glasses have you consumed on such a day?

Mark only one oval.

- 1
- 2
- 3
- 4
- 5
- 6
- 7
- 8
- 9
- 10
- 11
- 12

19. How often have you consumed whole breakfast grains in the last month? *

Attention: include grains only (milk and yoghurt will be inquired later on). Sum all grains you consumed on such a day: for example grains consumed during breakfast plus grains consumed as part of your dessert.

Mark only one oval.

- Not used
- 1 day in 4 weeks
- 2-3 days in 4 weeks
- 1 day per week
- 2 days per week
- 3 days per week
- 4 days per week
- 5 days per week
- 6 days per week
- 7 days per week

20. How many tablespoons have you consumed on such a day?

Mark only one oval.

- 1
- 2
- 3
- 4
- 5
- 6
- 7
- 8
- 9
- 10
- 11
- 12

21. What type of whole breakfast grains have you consumed on such a day? *

Mark only one oval per row.

	Rarely/never	Sometimes	Often	Usually/always
Whole porridge grains (brinta, oatmeal, etc.)	⬭	⬭	⬭	⬭
Whole grain muesli	⬭	⬭	⬭	⬭
Wheat bran	⬭	⬭	⬭	⬭

22. How often have you consumed other types of breakfast grains (cornflakes, cruesli, etc.) in the last month? *

Attention: include grains only (milk and yoghurt will be inquired later on). Sum all grains you consumed on such a day: for example grains consumed during breakfast plus grains consumed as part of your dessert.

Mark only one oval.

- ⬭ Not used
- ⬭ 1 day in 4 weeks
- ⬭ 2-3 days in 4 weeks
- ⬭ 1 day per week
- ⬭ 2 days per week
- ⬭ 3 days per week
- ⬭ 4 days per week
- ⬭ 5 days per week
- ⬭ 6 days per week
- ⬭ 7 days per week

23. How many tablespoons have you consumed on such a day?

Mark only one oval.

- 1
- 2
- 3
- 4
- 5
- 6
- 7
- 8
- 9
- 10
- 11
- 12

24. What type of other type of breakfast grains have you consumed on such a day? *

Mark only one oval per row.

	Rarely/never	Sometimes	Often	Usually/always
Refined porridge grains (semolina, custard, etc.)				
Cornflakes				
Crispy muesli, cruesli				
Fiber-rich breakfast products (All bran)				
Other types of breakfast products				

Bread and bread substitutes

25. How often have you consumed plain rusk, knäckebröd or breakfast crackers/crisp bread in the last month? *

Mark only one oval.

- Not used
- 1 day in 4 weeks
- 2-3 days in 4 weeks
- 1 day per week
- 2 days per week
- 3 days per week
- 4 days per week
- 5 days per week
- 6 days per week
- 7 days per week

26. How many pieces have you consumed on such a day?

Mark only one oval.

- 1
- 2
- 3
- 4
- 5
- 6
- 7
- 8
- 9
- 10
- 11
- 12

27. How often have you consumed croissants in the last month? *

Mark only one oval.

- Not used
- 1 day in 4 weeks
- 2-3 days in 4 weeks
- 1 day per week
- 2 days per week
- 3 days per week
- 4 days per week
- 5 days per week
- 6 days per week
- 7 days per week

28. How many pieces have you consumed on such a day?

Mark only one oval.

- 1
- 2
- 3
- 4
- 5
- 6
- 7
- 8
- 9
- 10
- 11
- 12

29. How often have you consumed gingerbread in the last month? *

Mark only one oval.

- Not used
- 1 day in 4 weeks
- 2-3 days in 4 weeks
- 1 day per week
- 2 days per week
- 3 days per week
- 4 days per week
- 5 days per week
- 6 days per week
- 7 days per week

30. How many pieces have you consumed on such a day?

Mark only one oval.

- 1
- 2
- 3
- 4
- 5
- 6
- 7
- 8
- 9
- 10
- 11
- 12

31. How much butter, margarine and/or halvarine spread have you consumed on plain rusk, knäckebröd, croissants or gingerbread? *

Mark only one oval.

- ( ) No spread
- ( ) Hardly covered with spread
- ( ) Covered with spread
- ( ) Generously covered with spread
- ( ) Very generously covered with spread

32. What type of butter, margarine and/or halvarine spread on plain rusk, knäckebröd, croissants or gingerbread have you consumed?

Tick all that apply.

	Plain rusk, knäckebröd or breakfast crackers/crisp bread	Croissants	Gingerbread
Halvarine or light margarine	☐	☐	☐
Diet halvarine or light diet margarine	☐	☐	☐
Halvarine with plant sterols/stanols	☐	☐	☐
Low-fat halvarine (25% fat or less)	☐	☐	☐
Margarine (tub)	☐	☐	☐
Margarine (package)	☐	☐	☐
Diet margarine	☐	☐	☐
Butter	☐	☐	☐
Semi-skimmed butter	☐	☐	☐

33. How often have you consumed rolls/buns in the last month? *

Mark only one oval.

- Not used
- 1 day in 4 weeks
- 2-3 days in 4 weeks
- 1 day per week
- 2 days per week
- 3 days per week
- 4 days per week
- 5 days per week
- 6 days per week
- 7 days per week

34. How many pieces have you consumed on such a day?

Mark only one oval.

- 1
- 2
- 3
- 4
- 5
- 6
- 7
- 8
- 9
- 10
- 11
- 12

35. What type of rolls/buns have you consumed on such a day? *

Mark only one oval per row.

	Rarely/never	Sometimes	Often	Usually/always
White rolls/buns	◯	◯	◯	◯
Brown rolls/buns	◯	◯	◯	◯
Whole grain rolls/buns	◯	◯	◯	◯
Multigrain rolls/buns	◯	◯	◯	◯
Raisin/muesli rolls/buns	◯	◯	◯	◯

36. How much butter, margarine and/or halvarine spread on rolls/buns have you consumed? *

Mark only one oval.

◯ No spread
◯ Hardly covered with spread
◯ Covered with spread
◯ Generously covered with spread
◯ Very generously covered with spread

37. What type of butter, margarine and/or halvarine spread on rolls/buns have you consumed?

Tick all that apply.

	Rolls/buns
Halvarine or light margarine	☐
Diet halvarine or light diet margarine	☐
Halvarine with plant sterols/stanols	☐
Low-fat halvarine (25% fat or less)	☐
Margarine (tub)	☐
Margarine (package)	☐
Diet margarine	☐
Butter	☐
Semi-skimmed butter	☐

38. How often have you consumed bread slices/sandwich bread in the last month? *

Mark only one oval.

- ◯ Not used
- ◯ 1 day in 4 weeks
- ◯ 2-3 days in 4 weeks
- ◯ 1 day per week
- ◯ 2 days per week
- ◯ 3 days per week
- ◯ 4 days per week
- ◯ 5 days per week
- ◯ 6 days per week
- ◯ 7 days per week

39. How many pieces have you consumed on such a day?

Mark only one oval.

- 1
- 2
- 3
- 4
- 5
- 6
- 7
- 8
- 9
- 10
- 11
- 12

40. What type of bread slices/sandwich bread have you consumed on such a day? *

Mark only one oval per row.

	Rarely/never	Sometimes	Often	Usually/always
White bread				
Brown bread				
Whole grain bread				
Multigrain bread				
Rye bread (light)				
Rye bread (dark)				
Raisin/muesli bread				

41. How much butter, margarine and/or halvarine spread on bread slices/sandwich bread have you consumed? *

Mark only one oval.

- No spread
- Hardly covered with spread
- Covered with spread
- Generously covered with spread
- Very generously covered with spread

42. What type of butter, margarine and/or halvarine spread on bread slices/sandwich bread have you consumed?

Tick all that apply.

	Bread slices/sandwich bread
Halvarine or light margarine	☐
Diet halvarine or light diet margarine	☐
Halvarine with plant sterols/stanols	☐
Low-fat halvarine (25% fat or less)	☐
Margarine (tub)	☐
Margarine (package)	☐
Diet margarine	☐
Butter	☐
Semi-skimmed butter	☐

Spreads

Attention: when you have two slices of bread putted on top of each other with spread on one slice of bread you count "1". When you put spread on both pieces and then put them on top of each other: count "2".

43. How often have you consumed soft cheeses or dairy spreads in the last month? *

For example: brie, camembert, cream cheese, etc.

Mark only one oval.

- Not used
- 1 day in 4 weeks
- 2-3 days in 4 weeks
- 1 day per week
- 2 days per week
- 3 days per week
- 4 days per week
- 5 days per week
- 6 days per week
- 7 days per week

44. How many servings have you consumed on such a day?

1 serving = enough to cover one slice of bread

Mark only one oval.

- 1
- 2
- 3
- 4
- 5
- 6
- 7
- 8
- 9
- 10
- 11
- 12

45. What type of cheese have you consumed on such a day? *

Mark only one oval per row.

	Rarely/never	Sometimes	Often	Usually/always
Processed cheese spread or dairy spread	◯	◯	◯	◯
Cream cheese or foreign cheeses	◯	◯	◯	◯

46. How often have you consumed hard cheeses in the last month? *

Mark only one oval.

- ◯ Not used
- ◯ 1 day in 4 weeks
- ◯ 2-3 days in 4 weeks
- ◯ 1 day per week
- ◯ 2 days per week
- ◯ 3 days per week
- ◯ 4 days per week
- ◯ 5 days per week
- ◯ 6 days per week
- ◯ 7 days per week

47. How many servings have you consumed on such a day?

1 serving = enough to cover one slice of bread

Mark only one oval.

- 1
- 2
- 3
- 4
- 5
- 6
- 7
- 8
- 9
- 10
- 11
- 12

48. What type of cheese have you consumed on such a day? *

Mark only one oval per row.

	Rarely/never	Sometimes	Often	Usually/always
20+ or 30+ cheese				
40+ or 48+ fatty cheese				

49. How much hard cheese have you consumed on one slice of bread?

Mark only one oval.

- One slice of bread is covered half
- One slice of bread is covered completely / presliced cheese / cheese sliced with a cheese slicer
- One slice of bread is covered generously / cheese sliced with knive / multiple layers of cheese sliced with cheese slicer

50. How often have you consumed cold cuts in the last month? *

Mark only one oval.

- Not used
- 1 day in 4 weeks
- 2-3 days in 4 weeks
- 1 day per week
- 2 days per week
- 3 days per week
- 4 days per week
- 5 days per week
- 6 days per week
- 7 days per week

51. How many servings have you consumed on such a day?

1 servings = enough to cover one slice of bread

Mark only one oval.

- 1
- 2
- 3
- 4
- 5
- 6
- 7
- 8
- 9
- 10
- 11
- 12

52. What type of cold cuts have you consumed on such a day? *

Mark only one oval per row.

	Rarely/never	Sometimes	Often	Usually/always
Filet americain	◯	◯	◯	◯
Boiled liver	◯	◯	◯	◯
Sausage: liver sausage, pate, liver pate, liver cheese	◯	◯	◯	◯
Ham: raw, smoked, boiled, processed	◯	◯	◯	◯
Roast beef, chicken breast	◯	◯	◯	◯
Sausage: cervelate, salami, etc.	◯	◯	◯	◯
Other types of culd cuts	◯	◯	◯	◯

53. How often have you consumed peanut butter or nut paste in the last month? *

Mark only one oval.

◯ Not used
◯ 1 day in 4 weeks
◯ 2-3 days in 4 weeks
◯ 1 day per week
◯ 2 days per week
◯ 3 days per week
◯ 4 days per week
◯ 5 days per week
◯ 6 days per week
◯ 7 days per week

54. How many servings have you consumed on such a day?

1 serving = enough to cover one slice of bread

Mark only one oval.

- 1
- 2
- 3
- 4
- 5
- 6
- 7
- 8
- 9
- 10
- 11
- 12

55. How often have you consumed sweet spreads in the last month? *

Mark only one oval.

- Not used
- 1 day in 4 weeks
- 2-3 days in 4 weeks
- 1 day per week
- 2 days per week
- 3 days per week
- 4 days per week
- 5 days per week
- 6 days per week
- 7 days per week

56. How many servings have you consumed on such a day?

1 serving = enough to cover one slice of bread

Mark only one oval.

- 1
- 2
- 3
- 4
- 5
- 6
- 7
- 8
- 9
- 10
- 11
- 12

57. What type of sweet spreads have you consumed on such a day? *

Mark only one oval per row.

	Rarely/never	Sometimes	Often	Usually/always
Chocolate sprinkles or flakes				
Chocolade paste, hazelnut paste, chocolate butter				
Other types of sweet spreads				

58. How often have you consumed sandwich spreads in the last month? *

Mark only one oval.

- Not used
- 1 day in 4 weeks
- 2-3 days in 4 weeks
- 1 day per week
- 2 days per week
- 3 days per week
- 4 days per week
- 5 days per week
- 6 days per week
- 7 days per week

59. How many servings have you consumed on such a day?

1 serving = enough to cover one slice of bread

Mark only one oval.

- 1
- 2
- 3
- 4
- 5
- 6
- 7
- 8
- 9
- 10
- 11
- 12

60. How often have you consumed salad spreads in the last month? *

Mark only one oval.

- Not used
- 1 day in 4 weeks
- 2-3 days in 4 weeks
- 1 day per week
- 2 days per week
- 3 days per week
- 4 days per week
- 5 days per week
- 6 days per week
- 7 days per week

61. How many servings have you consumed on such a day?

1 serving = enough to cover one slice of bread

Mark only one oval.

- 1
- 2
- 3
- 4
- 5
- 6
- 7
- 8
- 9
- 10
- 11
- 12

62. How often have you consumed yeast pasta (e.g. marmite) in the last month? *

Mark only one oval.

- Not used
- 1 day in 4 weeks
- 2-3 days in 4 weeks
- 1 day per week
- 2 days per week
- 3 days per week
- 4 days per week
- 5 days per week
- 6 days per week
- 7 days per week

63. How many servings have you consumed on such a day?

1 serving = enough to cover one slice of bread

Mark only one oval.

- 1
- 2
- 3
- 4
- 5
- 6
- 7
- 8
- 9
- 10
- 11
- 12

Eggs

64. How often have you consumed eggs in the last month? *

Mark only one oval.

- Not used
- 1 day in 4 weeks
- 2-3 days in 4 weeks
- 1 day per week
- 2 days per week
- 3 days per week
- 4 days per week
- 5 days per week
- 6 days per week
- 7 days per week

65. How many pieces have you consumed on such a day?

Mark only one oval.

- 1
- 2
- 3
- 4
- 5
- 6
- 7
- 8
- 9
- 10
- 11
- 12

66. How often did you boil or fry your eggs?

Mark only one oval.

- ( ) (Almost) always boiled
- ( ) (Almost) always fried
- ( ) Sometimes boiled, sometimes fried

67. If you fried your eggs, what type of butter, margarine and/or halvarine spread have you used?

Tick all that apply.

	Fried eggs
Halvarine or light margarine	☐
Diet halvarine or light diet margarine	☐
Halvarine with plant sterols/stanols	☐
Low-fat halvarine (25% fat or less)	☐
Margarine (tub)	☐
Margarine (package)	☐
Diet margarine	☐
Butter	☐
Semi-skimmed butter	☐

Dairy

Attention: please list dairy consumed with breakfast grains as well. Dairy in coffee and tea will be inquired later on.

68. How often have you consumed milk or buttermilk in the last month? *

Attention: dairy alternatives will be inquired later on.

Mark only one oval.

- Not used
- 1 day in 4 weeks
- 2-3 days in 4 weeks
- 1 day per week
- 2 days per week
- 3 days per week
- 4 days per week
- 5 days per week
- 6 days per week
- 7 days per week

69. How many glasses have you consumed on such a day?

Mark only one oval.

- 1
- 2
- 3
- 4
- 5
- 6
- 7
- 8
- 9
- 10
- 11
- 12

70. What type of milk have you consumed on such a day? *

Mark only one oval per row.

	Rarely/never	Sometimes	Often	Usually/always
Whole milk	⬭	⬭	⬭	⬭
Semi-skimmed milk	⬭	⬭	⬭	⬭
Skimmed milk	⬭	⬭	⬭	⬭
Buttermilk	⬭	⬭	⬭	⬭

71. How often have you consumed chocolate milk or sweetened milk drinks in the last month? *

Attention: dairy alternatives and health-improving dairy will be inquired later on.

Mark only one oval.

- ⬭ Not used
- ⬭ 1 day in 4 weeks
- ⬭ 2-3 days in 4 weeks
- ⬭ 1 day per week
- ⬭ 2 days per week
- ⬭ 3 days per week
- ⬭ 4 days per week
- ⬭ 5 days per week
- ⬭ 6 days per week
- ⬭ 7 days per week

72. How many glasses have you consumed on such a day?

Mark only one oval.

- 1
- 2
- 3
- 4
- 5
- 6
- 7
- 8
- 9
- 10
- 11
- 12

73. What type of sweetened milk drinks have you consumed on such a day? *

Mark only one oval per row.

	Rarely/never	Sometimes	Often	Usually/always
Chocolate milk				
Chocolate milk without sugar				
Sweetened yoghurt or milk drinks				
Yoghurt or milk drinks without sugar				

74. How often have you consumed animal-based dairy in the last month? *

For example: lactose-free milk or yoghurt, goat milk or yoghurt, sheep milk, etc.

Mark only one oval.

- Not used
- 1 day in 4 weeks
- 2-3 days in 4 weeks
- 1 day per week
- 2 days per week
- 3 days per week
- 4 days per week
- 5 days per week
- 6 days per week
- 7 days per week

75. How many glasses/bowls have you consumed on such a day?

Mark only one oval.

- 1
- 2
- 3
- 4
- 5
- 6
- 7
- 8
- 9
- 10
- 11
- 12

76. How often have you consumed dairy alternatives (plant-based) in the last month? *

For example: soy milk or yoghurt, rice milk, oat milk, coconut milk, almond milk, etc.

Mark only one oval.

- Not used
- 1 day in 4 weeks
- 2-3 days in 4 weeks
- 1 day per week
- 2 days per week
- 3 days per week
- 4 days per week
- 5 days per week
- 6 days per week
- 7 days per week

77. How many glasses/bowls have you consumed on such a day?

Mark only one oval.

- 1
- 2
- 3
- 4
- 5
- 6
- 7
- 8
- 9
- 10
- 11
- 12

78. What type of dairy alternatives have you consumed on such a day? *

Mark only one oval per row.

	Rarely/never	Sometimes	Often	Usually/always
Soy milk	⬭	⬭	⬭	⬭
Soy drink	⬭	⬭	⬭	⬭
Soy yoghurt or dessert	⬭	⬭	⬭	⬭

79. How often have you consumed health-improving dairy in the last month? *

For example: Yakult, Actimel, Activa, Vifit.

Mark only one oval.

- ⬭ Not used
- ⬭ 1 day in 4 weeks
- ⬭ 2-3 days in 4 weeks
- ⬭ 1 day per week
- ⬭ 2 days per week
- ⬭ 3 days per week
- ⬭ 4 days per week
- ⬭ 5 days per week
- ⬭ 6 days per week
- ⬭ 7 days per week

80. How many servings have you consumed on such a day?

1 serving = standard package/bottle

Mark only one oval.

- 1
- 2
- 3
- 4
- 5
- 6
- 7
- 8
- 9
- 10
- 11
- 12

81. How often have you consumed milkshake in the last month? *

Mark only one oval.

- Not used
- 1 day in 4 weeks
- 2-3 days in 4 weeks
- 1 day per week
- 2 days per week
- 3 days per week
- 4 days per week
- 5 days per week
- 6 days per week
- 7 days per week

82. How many glasses have you consumed on such a day?

Mark only one oval.

- 1
- 2
- 3
- 4
- 5
- 6
- 7
- 8
- 9
- 10
- 11
- 12

83. How often have you consumed dutch custard or pudding in the last month? *

Mark only one oval.

- Not used
- 1 day in 4 weeks
- 2-3 days in 4 weeks
- 1 day per week
- 2 days per week
- 3 days per week
- 4 days per week
- 5 days per week
- 6 days per week
- 7 days per week

84. How many bowls have you consumed on such a day?

Mark only one oval.

- 1
- 2
- 3
- 4
- 5
- 6
- 7
- 8
- 9
- 10
- 11
- 12

85. How often have you consumed (fruit) yoghurt or (fruit) quark in the last month? *

Mark only one oval.

- Not used
- 1 day in 4 weeks
- 2-3 days in 4 weeks
- 1 day per week
- 2 days per week
- 3 days per week
- 4 days per week
- 5 days per week
- 6 days per week
- 7 days per week

86. How many bowls have you consumed on such a day?

Mark only one oval.

- 1
- 2
- 3
- 4
- 5
- 6
- 7
- 8
- 9
- 10
- 11
- 12

87. What type of yoghurt or quark have you consumed on such a day? *

Mark only one oval per row.

	Rarely/never	Sometimes	Often	Usually/always
Whole (fruit) yoghurt/quark				
Semi-skimmed (fruit) yoghurt/quark				
Skimmed (fruit) yoghurt/quark				

88. How often have you consumed ready-made porridge in the last month? *

For example: oatmeal, semolina porridge, rice porridge.

Mark only one oval.

- Not used
- 1 day in 4 weeks
- 2-3 days in 4 weeks
- 1 day per week
- 2 days per week
- 3 days per week
- 4 days per week
- 5 days per week
- 6 days per week
- 7 days per week

89. How many bowls have you consumed on such a day?

Mark only one oval.

- 1
- 2
- 3
- 4
- 5
- 6
- 7
- 8
- 9
- 10
- 11
- 12

90. How often have you consumed (dairy-based) ice cream in the last month? *

Mark only one oval.

- Not used
- 1 day in 4 weeks
- 2-3 days in 4 weeks
- 1 day per week
- 2 days per week
- 3 days per week
- 4 days per week
- 5 days per week
- 6 days per week
- 7 days per week

91. How many bowls have you consumed on such a day?

1 cone = 1 bowl

Mark only one oval.

- 1
- 2
- 3
- 4
- 5
- 6
- 7
- 8
- 9
- 10
- 11
- 12

92. How often have you consumed whipped cream in the last month? *

Mark only one oval.

- Not used
- 1 day in 4 weeks
- 2-3 days in 4 weeks
- 1 day per week
- 2 days per week
- 3 days per week
- 4 days per week
- 5 days per week
- 6 days per week
- 7 days per week

93. How many tablespoons have you consumed on such a day?

Mark only one oval.

- 1-2
- 3-4
- 5-6
- 7-8
- 9-10
- 11-12
- 13-14
- 15-16
- 17-18
- 19-20

94. How often have you added sugar or syrup to your dairy products in the last month? *

Mark only one oval.

- Not used
- 1 day in 4 weeks
- 2-3 days in 4 weeks
- 1 day per week
- 2 days per week
- 3 days per week
- 4 days per week
- 5 days per week
- 6 days per week
- 7 days per week

95. How many teaspoons have you consumed on such a day?

Mark only one oval.

- 1
- 2
- 3
- 4
- 5
- 6
- 7
- 8
- 9
- 10
- 11
- 12

96. How often have you added honey to your dairy products in the last month? *

Mark only one oval.

- Not used
- 1 day in 4 weeks
- 2-3 days in 4 weeks
- 1 day per week
- 2 days per week
- 3 days per week
- 4 days per week
- 5 days per week
- 6 days per week
- 7 days per week

97. How many teaspoons have you consumed on such a day?

Mark only one oval.

- 1
- 2
- 3
- 4
- 5
- 6
- 7
- 8
- 9
- 10
- 11
- 12

98. How often have you added peanuts, nuts or seeds to your dairy products in the last month? *

Mark only one oval.

- Not used
- 1 day in 4 weeks
- 2-3 days in 4 weeks
- 1 day per week
- 2 days per week
- 3 days per week
- 4 days per week
- 5 days per week
- 6 days per week
- 7 days per week

99. How many servings have you consumed on such a day?

Mark only one oval.

- 1
- 2
- 3
- 4
- 5
- 6
- 7
- 8
- 9
- 10
- 11
- 12

100. What type of peanuts, nuts or seeds have you added on such a day? *

Mark only one oval per row.

	Rarely/never	Sometimes	Often	Usually/always
Peanuts	⬭	⬭	⬭	⬭
Nuts	⬭	⬭	⬭	⬭
Seeds	⬭	⬭	⬭	⬭

Coffee

101. How often have you consumed coffee in the last month? *

Mark only one oval.

- ⬭ Not used
- ⬭ 1 day in 4 weeks
- ⬭ 2-3 days in 4 weeks
- ⬭ 1 day per week
- ⬭ 2 days per week
- ⬭ 3 days per week
- ⬭ 4 days per week
- ⬭ 5 days per week
- ⬭ 6 days per week
- ⬭ 7 days per week

102. How many servings have you consumed on such a day?

Mark only one oval.

- 1
- 2
- 3
- 4
- 5
- 6
- 7
- 8
- 9
- 10
- 11
- 12

103. What type of coffee have you consumed on such a day? *

Mark only one oval per row.

	Rarely/never	Sometimes	Often	Usually/always
Decaffeinated coffee				
Filter coffee, percolator coffee, coffee pads, instant coffee				
Coffee made with french press (cafetière), boiled coffee, Greek of Turkish coffee				

104. How often have you added sugar to your coffee in the last month? *

Mark only one oval.

- Not used
- 1 day in 4 weeks
- 2-3 days in 4 weeks
- 1 day per week
- 2 days per week
- 3 days per week
- 4 days per week
- 5 days per week
- 6 days per week
- 7 days per week

105. How many servings have you consumed on such a day?

1 serving = 1 teaspoon or 1 sugar cube. Attention: sum all sugar consumed over the day. For example: if you drink 2 cups of coffee a day and in each cup you put 1 serving of sugar, the total amount of sugar consumed is 2 servings (fill out: 2 servings).

Mark only one oval.

- Minder dan 1
- 1
- 2
- 3
- 4
- 5
- 6
- 7
- 8
- 9
- 10
- 11
- 12
- Meer dan 12

106. How often have you added regular milk to your coffee in the last month? *

Mark only one oval.

- Not used
- 1 day in 4 weeks
- 2-3 days in 4 weeks
- 1 day per week
- 2 days per week
- 3 days per week
- 4 days per week
- 5 days per week
- 6 days per week
- 7 days per week

107. How many servings of coffee with milk added have you consumed on such a day?

Mark only one oval.

- 1
- 2
- 3
- 4
- 5
- 6
- 7
- 8
- 9
- 10
- 11
- 12

108. Have you usually used a cup or mug?

Mark only one oval.

- Cup
- Mug

109. If you added regular milk to your coffee, how much milk have you added to your coffee on such a day?

Mark only one oval.

- 3/4 part coffee, 1/4 part milk
- 1/2 part coffee, 1/2 part milk
- 1/4 part coffee, 3/4 part coffee
- Instant coffee resolved in milk (no water)

110. How often have you added coffee milk or creamer to your coffee in the last month? *

Mark only one oval.

- Not used
- 1 day in 4 weeks
- 2-3 days in 4 weeks
- 1 day per week
- 2 days per week
- 3 days per week
- 4 days per week
- 5 days per week
- 6 days per week
- 7 days per week

111. How many servings have you consumed on such a day?

Attention: sum all coffee milk or creamer consumed over the day. For example: if you drink 2 cups of coffee a day and in each cup you put 1 serving of coffee creamer, the total amount of coffee creamer consumed is 2 servings (fill out: 2 servings).

Mark only one oval.

- Minder dan 1
- 1
- 2
- 3
- 4
- 5
- 6
- 7
- 8
- 9
- 10
- 11
- 12
- Meer dan 12

Tea

112. How often have you consumed tea in the last month? *

Mark only one oval.

- Not used
- 1 day in 4 weeks
- 2-3 days in 4 weeks
- 1 day per week
- 2 days per week
- 3 days per week
- 4 days per week
- 5 days per week
- 6 days per week
- 7 days per week

113. How many servings have you consumed on such a day?

Mark only one oval.

- 1
- 2
- 3
- 4
- 5
- 6
- 7
- 8
- 9
- 10
- 11
- 12

114. What type of tea have you consumed on such a day? *

Mark only one oval per row.

	Rarely/never	Sometimes	Often	Usually/always
Black tea	◯	◯	◯	◯
Green tea	◯	◯	◯	◯
Herbal tea	◯	◯	◯	◯

115. How often have you added sugar to your tea in the last month? *

Mark only one oval.

- ◯ Not used
- ◯ 1 day in 4 weeks
- ◯ 2-3 days in 4 weeks
- ◯ 1 day per week
- ◯ 2 days per week
- ◯ 3 days per week
- ◯ 4 days per week
- ◯ 5 days per week
- ◯ 6 days per week
- ◯ 7 days per week

116. How many servings have you consumed on such a day?

1 serving = 1 teaspoon or 1 sugar cube. Attention: sum all sugar consumed over the day. For example: if you drink 2 cups of tea a day and in each cup you put 1 serving of sugar, the total amount of sugar consumed is 2 servings (fill out: 2 servings).

Mark only one oval.

- Minder dan 1
- 1
- 2
- 3
- 4
- 5
- 6
- 7
- 8
- 9
- 10
- 11
- 12
- Meer dan 12

117. How often have you added honey to your tea in the last month? *

Mark only one oval.

- Not used
- 1 day in 4 weeks
- 2-3 days in 4 weeks
- 1 day per week
- 2 days per week
- 3 days per week
- 4 days per week
- 5 days per week
- 6 days per week
- 7 days per week

118. How many servings have you consumed on such a day?

1 serving = 1 teaspoon. Attention: sum all honey consumed over the day. For example: if you drink 2 cups of tea a day and in each cup you put 1 serving of honey, the total amount of honey consumed is 2 servings (fill out: 2 servings).

Mark only one oval.

- Minder dan 1
- 1
- 2
- 3
- 4
- 5
- 6
- 7
- 8
- 9
- 10
- 11
- 12
- Meer dan 12

Dinner

The following questions are about warm meals usually eaten as dinner. It is possible that you consume these products at another timepoint on the day, for example during breakfast, lunch or as snack. Please include the consumption of these timepoints as well. Attention: when you get multiple portions, please sum and note the totale amount you have consumed.

119. How often have you consumed soup in the last month? *

Mark only one oval.

- Not used
- 1 day in 4 weeks
- 2-3 days in 4 weeks
- 1 day per week
- 2 days per week
- 3 days per week
- 4 days per week
- 5 days per week
- 6 days per week
- 7 days per week

120. How many bowls have you consumed on such a day?

Mark only one oval.

- 1
- 2
- 3
- 4
- 5
- 6
- 7
- 8
- 9
- 10
- 11
- 12

121. What type of soup have you consumed on such a day? *

Mark only one oval per row.

	Rarely/never	Sometimes	Often	Usually/always
Soup with legumes	◯	◯	◯	◯
Soup without legumes	◯	◯	◯	◯

122. How often have you consumed pizza in the last month? *

Mark only one oval.

- ◯ Not used
- ◯ 1 day in 4 weeks
- ◯ 2-3 days in 4 weeks
- ◯ 1 day per week
- ◯ 2 days per week
- ◯ 3 days per week
- ◯ 4 days per week
- ◯ 5 days per week
- ◯ 6 days per week
- ◯ 7 days per week

123. How many pizzas have you consumed on such a day?

Mark only one oval.

- ◯ 0.5
- ◯ 1
- ◯ 1.5
- ◯ 2
- ◯ 2.5
- ◯ 3

124. How often have you consumed pancakes in the last month? *

Mark only one oval.

- Not used
- 1 day in 4 weeks
- 2-3 days in 4 weeks
- 1 day per week
- 2 days per week
- 3 days per week
- 4 days per week
- 5 days per week
- 6 days per week
- 7 days per week

125. How many pancakes have you consumed on such a day?

Mark only one oval.

- 1
- 2
- 3
- 4
- 5
- 6
- 7
- 8
- 9
- 10
- 11
- 12

126. What size was your pancake usually?

Mark only one oval.

- Size of a breakfast plate
- Size of a regular dinner plate
- Size of a XL dinner plate

127. How often have you consumed ready-made meals in the last month? *

Mark only one oval.

- Not used
- 1 day in 4 weeks
- 2-3 days in 4 weeks
- 1 day per week
- 2 days per week
- 3 days per week
- 4 days per week
- 5 days per week
- 6 days per week
- 7 days per week

128. How many serving spoons have you consumed on such a day?

One ready-made meal is approximately 8 serving spoons.

Mark only one oval.

- 1
- 2
- 3
- 4
- 5
- 6
- 7
- 8
- 9
- 10
- 11
- 12

129. What type of ready-made meal have you consumed on such a day? *

Mark only one oval per row.

	Rarely/never	Sometimes	Often	Usually/always
Dutch meal				
Italian meal				
Oriental meal				
Other types of ready-made meals				

130. How often have you consumed pasta in the last month? *

Mark only one oval.

- Not used
- 1 day in 4 weeks
- 2-3 days in 4 weeks
- 1 day per week
- 2 days per week
- 3 days per week
- 4 days per week
- 5 days per week
- 6 days per week
- 7 days per week

131. How many serving spoons have you consumed on such a day?

Attention: pasta only; do not take into account sauces/vegetables.

Mark only one oval.

- 1
- 2
- 3
- 4
- 5
- 6
- 7
- 8
- 9
- 10
- 11
- 12

132. What type of pasta have you consumed on such a day? *

Mark only one oval per row.

	Rarely/never	Sometimes	Often	Usually/always
White pasta	◯	◯	◯	◯
Whole grain pasta	◯	◯	◯	◯

133. How often have you consumed rice in the last month? *

Mark only one oval.

- ◯ Not used
- ◯ 1 day in 4 weeks
- ◯ 2-3 days in 4 weeks
- ◯ 1 day per week
- ◯ 2 days per week
- ◯ 3 days per week
- ◯ 4 days per week
- ◯ 5 days per week
- ◯ 6 days per week
- ◯ 7 days per week

134. How many serving spoons have you consumed on such a day?

Attention: rice only; do not take into account sauces/vegetables.

Mark only one oval.

- 1
- 2
- 3
- 4
- 5
- 6
- 7
- 8
- 9
- 10
- 11
- 12

135. What type of rice have you consumed on such a day? *

Mark only one oval per row.

	Rarely/never	Sometimes	Often	Usually/always
White rice				
Whole grain rice				

136. How often have you consumed grains in the last month? *

For example: bulgur, couscous, millet, etc.

Mark only one oval.

- Not used
- 1 day in 4 weeks
- 2-3 days in 4 weeks
- 1 day per week
- 2 days per week
- 3 days per week
- 4 days per week
- 5 days per week
- 6 days per week
- 7 days per week

137. How many serving spoons have you consumed on such a day?

Attention: grains only; do not take into account sauces/vegetables.

Mark only one oval.

- 1
- 2
- 3
- 4
- 5
- 6
- 7
- 8
- 9
- 10
- 11
- 12

138. How often have you consumed legumes in the last month? *

For example: brown, white, black beans, capuchin, lentils, etc. Peas, green beans and broad beans will be inquired later on.

Mark only one oval.

- Not used
- 1 day in 4 weeks
- 2-3 days in 4 weeks
- 1 day per week
- 2 days per week
- 3 days per week
- 4 days per week
- 5 days per week
- 6 days per week
- 7 days per week

139. How many serving spoons have you consumed on such a day?

Mark only one oval.

- 1
- 2
- 3
- 4
- 5
- 6
- 7
- 8
- 9
- 10
- 11
- 12

Potatoes

140. How often have you consumed boiled and mashed potatoes in the last month?
*

Mark only one oval.

- Not used
- 1 day in 4 weeks
- 2-3 days in 4 weeks
- 1 day per week
- 2 days per week
- 3 days per week
- 4 days per week
- 5 days per week
- 6 days per week
- 7 days per week

141. How many serving spoons have you consumed on such a day?

Mark only one oval.

- 1
- 2
- 3
- 4
- 5
- 6
- 7
- 8
- 9
- 10
- 11
- 12

142. How often have you consumed fries or baked or fried potatoes in the last month? *

Mark only one oval.

- Not used
- 1 day in 4 weeks
- 2-3 days in 4 weeks
- 1 day per week
- 2 days per week
- 3 days per week
- 4 days per week
- 5 days per week
- 6 days per week
- 7 days per week

143. How many serving spoons have you consumed on such a day?

Mark only one oval.

- 1
- 2
- 3
- 4
- 5
- 6
- 7
- 8
- 9
- 10
- 11
- 12

144. What type of potatoes have you consumed on such a day? *

Mark only one oval per row.

	Rarely/never	Sometimes	Often	Usually/always
Fries	⬭	⬭	⬭	⬭
Fried or baked potatoes, croquettes, rösti, etc.	⬭	⬭	⬭	⬭

145. What type of cooking oils/fats have you used to prepare baked or fried potatoes?

Tick all that apply.

	Baked/fried potatoes or fries
Margarine (tub)	☐
Margarine (package)	☐
Diet margarine	☐
Butter	☐
Backing and roasting product (solid)	☐
Backing and roasting product (liquid)	☐
Frying fat (solid)	☐
Frying fat (liquid)	☐
Olive oil	☐
Sunflower, soy or salad oil	☐
Bacon or beef fat	☐
Coconut oil	☐
No cooking oil/fat	☐

Vegetables

146. How often have you consumed boiled or stir-fried union in the last month? *

Mark only one oval.

- Not used
- 1 day in 4 weeks
- 2-3 days in 4 weeks
- 1 day per week
- 2 days per week
- 3 days per week
- 4 days per week
- 5 days per week
- 6 days per week
- 7 days per week

147. How many tablespoons have you consumed on such a day?

Mark only one oval.

- 1
- 2
- 3
- 4
- 5
- 6
- 7
- 8
- 9
- 10
- 11
- 12

148. How often have you consumed boiled or stir-fried vegetables in the last month? *

Attention: include vegetables used in mixed dishes as well.

Mark only one oval.

- Not used
- 1 day in 4 weeks
- 2-3 days in 4 weeks
- 1 day per week
- 2 days per week
- 3 days per week
- 4 days per week
- 5 days per week
- 6 days per week
- 7 days per week

149. How many tablespoons have you consumed on such a day?

Mark only one oval.

- 1
- 2
- 3
- 4
- 5
- 6
- 7
- 8
- 9
- 10
- 11
- 12

150. What type of boiled or stir-fried vegetables have you consumed on such a day? *

Mark only one oval per row.

	Not used	Less than 1 day per month	1 day per month	2-3 days per month	1 day per week	2-3 days per week	4-5 days per week	6-7 days per week
Endive								
Beetroot								
Cauliflower								
Broccoli								
Mushrooms								
Bell peppers								
Leek								
Red cabbage								
Green beans, string beans, broad beans								
Spinach								
Tomato								
Belgian endive/chicory								
White cabbage, pointed cabbage, green cabbage, savoy cabbage, Chinese cabbage, sauerkraut, kale, Brussel sprouts								
Carrot								
Other types of								

boiled or stir-fried vegetables

151. How have you prepared your vegetables usually?

Mark only one oval.

- ( ) Boiled, served with butter or margarine
- ( ) Boiled, served without butter or margarine
- ( ) Stir-fried
- ( ) Sometimes boiled and served without butter, sometimes boiled and served with butter or sometimes stir-fried

152. What type of cooking oils/fats have you used to prepare vegetables?

Tick all that apply.

	Baked/fried potatoes or fries
Margarine (tub)	☐
Margarine (package)	☐
Diet margarine	☐
Butter	☐
Backing and roasting product (solid)	☐
Backing and roasting product (liquid)	☐
Frying fat (solid)	☐
Frying fat (liquid)	☐
Olive oil	☐
Sunflower, soy or salad oil	☐
Bacon or beef fat	☐
Coconut oil	☐
No cooking oil/fat	☐

153. How often have you consumed raw vegetables in the last month? *

Attention: include vegetables used in salads/lunch as well.

Mark only one oval.

- Not used
- 1 day in 4 weeks
- 2-3 days in 4 weeks
- 1 day per week
- 2 days per week
- 3 days per week
- 4 days per week
- 5 days per week
- 6 days per week
- 7 days per week

154. How many bowls have you consumed on such a day?

Mark only one oval.

- 1
- 2
- 3
- 4
- 5
- 6
- 7
- 8
- 9
- 10
- 11
- 12

155. What type of raw vegetables have you consumed on such a day? *

Mark only one oval per row.

	Not used	Less than 1 day per month	1 day per month	2-3 days per month	1 day per week	2-3 days per week	4-5 days per week	6-7 days per week
Lettuce	◯	◯	◯	◯	◯	◯	◯	◯
Cucumber	◯	◯	◯	◯	◯	◯	◯	◯
Cabbage	◯	◯	◯	◯	◯	◯	◯	◯
Bell peppers	◯	◯	◯	◯	◯	◯	◯	◯
Tomato	◯	◯	◯	◯	◯	◯	◯	◯
Carrot	◯	◯	◯	◯	◯	◯	◯	◯
Union	◯	◯	◯	◯	◯	◯	◯	◯
Other raw vegetables	◯	◯	◯	◯	◯	◯	◯	◯

156. How often have you consumed dressing with your raw vegetables in the last month? *

Attention: include dressings used in other dishes as well.

Mark only one oval.

- ◯ Not used
- ◯ 1 day in 4 weeks
- ◯ 2-3 days in 4 weeks
- ◯ 1 day per week
- ◯ 2 days per week
- ◯ 3 days per week
- ◯ 4 days per week
- ◯ 5 days per week
- ◯ 6 days per week
- ◯ 7 days per week

157. How many tablespoons have you consumed on such a day?

Mark only one oval.

- Less than 0.5
- 0.5
- 1
- 1.5
- 2
- 2.5
- 3
- More than 3

158. What type of dressing have you consumed on such a day? *

Mark only one oval per row.

	Rarely/never	Sometimes	Often	Usually/always
Dressing or salad dressing				
Clear dressing without oil				

Fish, shellfish and crustaceans

159. How often have you consumed fish in the last month? *

Mark only one oval.

- Not used
- 1 day in 4 weeks
- 2-3 days in 4 weeks
- 1 day per week
- 2 days per week
- 3 days per week
- 4 days per week
- 5 days per week
- 6 days per week
- 7 days per week

160. How many servings have you consumed on such a day?

1 serving = standard portion of fried haddock, 1 salty herring or one piece of trout or salmon.

Mark only one oval.

- Less than 0.5
- 0.5
- 1
- 1.5
- 2
- 2.5
- 3
- More than 3

161. What type of fish have you consumed on such a day? *

Mark only one oval per row.

	Rarely/never	Sometimes	Often	Usually/always
Fried haddock or fried cod fish	◯	◯	◯	◯
Fish sticks	◯	◯	◯	◯
Lean fish: cod, plaice, tilapia, pangasius, trout, tuna, etc.	◯	◯	◯	◯
Fatty fish: salmon, mackerel, eel, pan herring, etc.	◯	◯	◯	◯
Salty herring	◯	◯	◯	◯

162. How have you prepared your fish usually?

Mark only one oval.

◯ Prepared, backed or fried, with fat
◯ Prepared without fat (include salty herring and smoked fish)
◯ Sometimes prepared with fat, sometimes prepared without fat

163. What type of cooking oils/fats have you used to prepare fish?

Tick all that apply.

	Baked/fried potatoes or fries
Margarine (tub)	☐
Margarine (package)	☐
Diet margarine	☐
Butter	☐
Backing and roasting product (solid)	☐
Backing and roasting product (liquid)	☐
Frying fat (solid)	☐
Frying fat (liquid)	☐
Olive oil	☐
Sunflower, soy or salad oil	☐
Bacon or beef fat	☐
Coconut oil	☐
No cooking oil/fat	☐

164. How often have you consumed shellfish and crustaceans in the last month? *

For example: mussels, shrimps/prawns, squid, escargot, etc.

Mark only one oval.

- Not used
- 1 day in 4 weeks
- 2-3 days in 4 weeks
- 1 day per week
- 2 days per week
- 3 days per week
- 4 days per week
- 5 days per week
- 6 days per week
- 7 days per week

165. How many servings have you consumed on such a day?

1 serving = e.g. 1kg mussels with shell or 240 gram without shell, or 100 gram shrimps/prawns meaning 7 big prawns or 7 tablespoons little shrimps.

Mark only one oval.

- Less than 0.5
- 0.5
- 1
- 1.5
- 2
- 2.5
- 3
- More than 3

166. What type of shellfish and crustaceans have you consumed on such a day? *

Mark only one oval per row.

	Rarely/never	Sometimes	Often	Usually/always
Shrimps or prawns	⬭	⬭	⬭	⬭
Mussels	⬭	⬭	⬭	⬭
Other types of shellfish or crustaceans	⬭	⬭	⬭	⬭

Meat and meat substitutes

167. How often have you consumed beef in the last month? *

Attention: do not include minced meat, this will be inquired later on.

Mark only one oval.

- Not used
- 1 day in 4 weeks
- 2-3 days in 4 weeks
- 1 day per week
- 2 days per week
- 3 days per week
- 4 days per week
- 5 days per week
- 6 days per week
- 7 days per week

168. How many servings have you consumed on such a day?

1 serving = e.g. 1 piece tartar, 1 sausage, 1 steak or 100 grams of other types of meat.

Mark only one oval.

- Less than 0.5
- 0.5
- 1
- 1.5
- 2
- 2.5
- 3
- More than 3

169. What type of beef have you consumed on such a day? *

Mark only one oval per row.

	Rarely/never	Sometimes	Often	Usually/always
Rump steak, beef steak, tartar, roasted beef				
Prime rib, braising steak, rib chop steak				
Seasoned, breaded or processed: beef roulade, beef olives				

170. How often have you consumed pork in the last month? *

Mark only one oval.

- Not used
- 1 day in 4 weeks
- 2-3 days in 4 weeks
- 1 day per week
- 2 days per week
- 3 days per week
- 4 days per week
- 5 days per week
- 6 days per week
- 7 days per week

171. How many servings have you consumed on such a day?

1 serving = e.g. 1 pork olive, 1 bacon chop or 100 grams of other type of meat.

Mark only one oval.

- Less than 0.5
- 0.5
- 1
- 1.5
- 2
- 2.5
- 3
- More than 3

172. What type of pork have you consumed on such a day? *

Mark only one oval per row.

	Rarely/never	Sometimes	Often	Usually/always
Bacon	⬭	⬭	⬭	⬭
Pork tenderloins, etc.	⬭	⬭	⬭	⬭
Pork chops, etc.	⬭	⬭	⬭	⬭
Seasoned, breaded or processed: schnitzel, spareribs, etc.	⬭	⬭	⬭	⬭

173. How often have you consumed poultry in the last month? *

Mark only one oval.

- Not used
- 1 day in 4 weeks
- 2-3 days in 4 weeks
- 1 day per week
- 2 days per week
- 3 days per week
- 4 days per week
- 5 days per week
- 6 days per week
- 7 days per week

174. How many servings have you consumed on such a day?

Mark only one oval.

- Less than 0.5
- 0.5
- 1
- 1.5
- 2
- 2.5
- 3
- More than 3

175. What type of poultry have you consumed on such a day? *

Mark only one oval per row.

	Rarely/never	Sometimes	Often	Usually/always
Chicken or other type of poultry				
Seasoned, breaded or processed: chicken nuggets, chicken burger, chicken schnitzel, etc.				

176. How often have you consumed other types of meat in the last month? *

Mark only one oval.

- Not used
- 1 day in 4 weeks
- 2-3 days in 4 weeks
- 1 day per week
- 2 days per week
- 3 days per week
- 4 days per week
- 5 days per week
- 6 days per week
- 7 days per week

177. How many servings have you consumed on such a day?

Mark only one oval.

- Less than 0.5
- 0.5
- 1
- 1.5
- 2
- 2.5
- 3
- More than 3

178. What type of other types of meat have you consumed on such a day? *

Mark only one oval per row.

	Rarely/never	Sometimes	Often	Usually/always
Minced meat	◯	◯	◯	◯
Hamburger	◯	◯	◯	◯
Sausages (beef, pork, etc)	◯	◯	◯	◯
Liver	◯	◯	◯	◯
Other types of meat or game	◯	◯	◯	◯

179. How often have you consumed meat substitutes in the last month? *

For example: tofu, quorn, valess.

Mark only one oval.

- ◯ Not used
- ◯ 1 day in 4 weeks
- ◯ 2-3 days in 4 weeks
- ◯ 1 day per week
- ◯ 2 days per week
- ◯ 3 days per week
- ◯ 4 days per week
- ◯ 5 days per week
- ◯ 6 days per week
- ◯ 7 days per week

180. How many servings have you consumed on such a day?

Mark only one oval.

- Less than 0.5
- 0.5
- 1
- 1.5
- 2
- 2.5
- 3
- More than 3

181. What type of cooking oils/fats have you used to prepare meat and/or meat substitutes?

Tick all that apply.

	Meat	Meat substitutes
Margarine (tub)	☐	☐
Margarine (package)	☐	☐
Diet margarine	☐	☐
Butter	☐	☐
Backing and roasting product (solid)	☐	☐
Backing and roasting product (liquid)	☐	☐
Frying fat (solid)	☐	☐
Frying fat (liquid)	☐	☐
Olive oil	☐	☐
Sunflower, soy or salad oil	☐	☐
Bacon or beef fat	☐	☐
Coconut oil	☐	☐
No cooking oil/fat	☐	☐

182. How often have you consumed gravy in the last month? *

Mark only one oval.

- Not used
- 1 day in 4 weeks
- 2-3 days in 4 weeks
- 1 day per week
- 2 days per week
- 3 days per week
- 4 days per week
- 5 days per week
- 6 days per week
- 7 days per week

183. How many gravy spoons have you consumed on such a day?

Mark only one oval.

- 1
- 2
- 3
- 4
- 5
- 6
- 7
- 8
- 9
- 10
- 11
- 12

184. What type of spoon was usually used for gravy?

Mark only one oval.

- Tablespoon
- Flat gravy spoon (belongs to cutlery usually)
- Deep gravy spoon (hangs on spoon rack usually)
- Unknown size of spoon

185. How many parts of the gravy were water?

Mark only one oval.

- No water, only backing product
- 1/4 water, 3/4 backing product
- 1/2 water, 1/2 backing product
- 3/4 water, 1/2 backing product
- Unknown

186. What type of cooking oils/fats have you used to prepare gravy?

Tick all that apply.

	Gravy
Margarine (tub)	☐
Margarine (package)	☐
Diet margarine	☐
Butter	☐
Backing and roasting product (solid)	☐
Backing and roasting product (liquid)	☐
Frying fat (solid)	☐
Frying fat (liquid)	☐
Olive oil	☐
Sunflower, soy or salad oil	☐
Bacon or beef fat	☐
Coconut oil	☐
No cooking oil/fat	☐

187. How often have you consumed warm sauces in the last month? *

For example: tomato sauce, satay sauce, bechamel sauce, etc.

Mark only one oval.

- Not used
- 1 day in 4 weeks
- 2-3 days in 4 weeks
- 1 day per week
- 2 days per week
- 3 days per week
- 4 days per week
- 5 days per week
- 6 days per week
- 7 days per week

188. How many sauce ladles have you consumed on such a day?

Mark only one oval.

- 1
- 2
- 3
- 4
- 5
- 6
- 7
- 8
- 9
- 10
- 11
- 12

189. What type of warm sauces have you consumed on such a day? *

Mark only one oval per row.

	Rarely/never	Sometimes	Often	Usually/always
Satay sauce	◯	◯	◯	◯
Tomato sauce	◯	◯	◯	◯
Other types of sauces	◯	◯	◯	◯

190. How often have you consumed mayonnaise, ketchup or other cold sauces at dinner in the last month? *

Mark only one oval.

- ◯ Not used
- ◯ 1 day in 4 weeks
- ◯ 2-3 days in 4 weeks
- ◯ 1 day per week
- ◯ 2 days per week
- ◯ 3 days per week
- ◯ 4 days per week
- ◯ 5 days per week
- ◯ 6 days per week
- ◯ 7 days per week

191. How many tablespoons have you consumed on such a day?

Mark only one oval.

- 1
- 2
- 3
- 4
- 5
- 6
- 7
- 8
- 9
- 10
- 11
- 12

192. What type of cold sauces have you consumed on such a day? *

Mark only one oval per row.

	Rarely/never	Sometimes	Often	Usually/always
Mayonnaise				
Halvanaise, fries sauce and other non-red sauces				
Tomato ketchup and other red sauces				

193. How often have you consumed mosterd (added to dinner or snacks) in the last month? *

Mark only one oval.

- Not used
- 1 day in 4 weeks
- 2-3 days in 4 weeks
- 1 day per week
- 2 days per week
- 3 days per week
- 4 days per week
- 5 days per week
- 6 days per week
- 7 days per week

194. How many teaspoons have you consumed on such a day?

Mark only one oval.

- 1
- 2
- 3
- 4
- 5
- 6
- 7
- 8
- 9
- 10
- 11
- 12

195. How often have you consumed sambal (added to dinner or snacks) in the last month? *

Mark only one oval.

- Not used
- 1 day in 4 weeks
- 2-3 days in 4 weeks
- 1 day per week
- 2 days per week
- 3 days per week
- 4 days per week
- 5 days per week
- 6 days per week
- 7 days per week

196. How many teaspoons have you consumed on such a day?

Mark only one oval.

- Less than 0.5
- 0.5
- 1
- 1.5
- 2
- 2.5
- 3
- More than 3

Additions to dinner

197. How often have you consumed nuts or seeds in the last month? *

For example: cashew nuts, pine nuts, sunflower seeds.

Mark only one oval.

- Not used
- 1 day in 4 weeks
- 2-3 days in 4 weeks
- 1 day per week
- 2 days per week
- 3 days per week
- 4 days per week
- 5 days per week
- 6 days per week
- 7 days per week

198. How many tablespoons have you consumed on such a day?

Mark only one oval.

- 1
- 2
- 3
- 4
- 5
- 6
- 7
- 8
- 9
- 10
- 11
- 12

199. What type of nuts have you consumed on such a day? *

Mark only one oval per row.

	Rarely/never	Sometimes	Often	Usually/always
Nuts, nut mix, trail mix	◯	◯	◯	◯
Seeds	◯	◯	◯	◯

200. How often have you consumed cheese added to dinner in the last month? *

For example: grated cheese, cheese cubes, feta.

Mark only one oval.

- ◯ Not used
- ◯ 1 day in 4 weeks
- ◯ 2-3 days in 4 weeks
- ◯ 1 day per week
- ◯ 2 days per week
- ◯ 3 days per week
- ◯ 4 days per week
- ◯ 5 days per week
- ◯ 6 days per week
- ◯ 7 days per week

201. How many tablespoons have you consumed on such a day?

Calculate 15 spoons for cheese fondue.

Mark only one oval.

- 1-2
- 3-4
- 5-6
- 7-8
- 9-10
- 11-12
- 13-14
- 15-16
- 17-18
- 19-20

202. How often have you consumed cream added to dinner in the last month? *

For example: creme fraiche, sour cream, whipped cream, cooking cream.

Mark only one oval.

- Not used
- 1 day in 4 weeks
- 2-3 days in 4 weeks
- 1 day per week
- 2 days per week
- 3 days per week
- 4 days per week
- 5 days per week
- 6 days per week
- 7 days per week

203. How many tablespoons have you consumed on such a day?

Mark only one oval.

- 1
- 2
- 3
- 4
- 5
- 6
- 7
- 8
- 9
- 10
- 11
- 12

Fruit

204. How often have you consumed citrus fruits in the last month? *

Mark only one oval.

- Not used
- 1 day in 4 weeks
- 2-3 days in 4 weeks
- 1 day per week
- 2 days per week
- 3 days per week
- 4 days per week
- 5 days per week
- 6 days per week
- 7 days per week

205. How many pieces have you consumed on such a day?

Mark only one oval.

- 1
- 2
- 3
- 4
- 5
- 6
- 7
- 8
- 9
- 10
- 11
- 12

206. What type of citrus fruits have you consumed on such a day? *

Mark only one oval per row.

	Not used	Less than 1 day per month	1 day per month	2-3 days per month	1 day per week	2-3 days per week	4-5 days per week	6-7 days per week
Mandarine								
Orange								
Grapefruit								
Other types of citrus fruit								

207. How often have you consumed other fruits in the last month? *

Mark only one oval.

- Not used
- 1 day in 4 weeks
- 2-3 days in 4 weeks
- 1 day per week
- 2 days per week
- 3 days per week
- 4 days per week
- 5 days per week
- 6 days per week
- 7 days per week

208. How many pieces have you consumed on such a day?

Mark only one oval.

- 1
- 2
- 3
- 4
- 5
- 6
- 7
- 8
- 9
- 10
- 11
- 12

209. What type of citrus fruits have you consumed on such a day? *

Mark only one oval per row.

	Not used	Less than 1 day per month	1 day per month	2-3 days per month	1 day per week	2-3 days per week	4-5 days per week	6-7 days per week
Strawberries	◯	◯	◯	◯	◯	◯	◯	◯
Apple	◯	◯	◯	◯	◯	◯	◯	◯
Banana	◯	◯	◯	◯	◯	◯	◯	◯
Berries	◯	◯	◯	◯	◯	◯	◯	◯
Grapes	◯	◯	◯	◯	◯	◯	◯	◯
Cherries	◯	◯	◯	◯	◯	◯	◯	◯
Kiwi	◯	◯	◯	◯	◯	◯	◯	◯
Melon	◯	◯	◯	◯	◯	◯	◯	◯
Pear	◯	◯	◯	◯	◯	◯	◯	◯
Other fruits	◯	◯	◯	◯	◯	◯	◯	◯

210. How often have you consumed dried fruits in the last month? *

Mark only one oval.

- ◯ Not used
- ◯ 1 day in 4 weeks
- ◯ 2-3 days in 4 weeks
- ◯ 1 day per week
- ◯ 2 days per week
- ◯ 3 days per week
- ◯ 4 days per week
- ◯ 5 days per week
- ◯ 6 days per week
- ◯ 7 days per week

211. How many servings have you consumed on such a day?

Mark only one oval.

- 1
- 2
- 3
- 4
- 5
- 6
- 7
- 8
- 9
- 10
- 11
- 12

Sweet snacks

212. How often have you consumed small biscuits or cookies in the last month? *

For example: gingerbread cookies, bastogne cookies, vanilla pretzel cookies.

Mark only one oval.

- Not used
- 1 day in 4 weeks
- 2-3 days in 4 weeks
- 1 day per week
- 2 days per week
- 3 days per week
- 4 days per week
- 5 days per week
- 6 days per week
- 7 days per week

213. How many pieces have you consumed on such a day?

Mark only one oval.

- 1
- 2
- 3
- 4
- 5
- 6
- 7
- 8
- 9
- 10
- 11
- 12

214. How often have you consumed nutritional biscuits or muesli bars in the last month? *

Mark only one oval.

- Not used
- 1 day in 4 weeks
- 2-3 days in 4 weeks
- 1 day per week
- 2 days per week
- 3 days per week
- 4 days per week
- 5 days per week
- 6 days per week
- 7 days per week

215. How many pieces have you consumed on such a day?

Sometimes a packing includes two biscuits. When you eat them both, note 2 servings.

Mark only one oval.

- 1
- 2
- 3
- 4
- 5
- 6
- 7
- 8
- 9
- 10
- 11
- 12

216. How often have you consumed big cookies or cake in the last month? *

For example: stroopwafel cookies, stuffed cookies, butter cake, Dutch egg cake.

Mark only one oval.

- Not used
- 1 day in 4 weeks
- 2-3 days in 4 weeks
- 1 day per week
- 2 days per week
- 3 days per week
- 4 days per week
- 5 days per week
- 6 days per week
- 7 days per week

217. How many pieces have you consumed on such a day?

Sometimes a packing includes two biscuits. When you eat them both, note 2 servings.

Mark only one oval.

- ( ) 1
- ( ) 2
- ( ) 3
- ( ) 4
- ( ) 5
- ( ) 6
- ( ) 7
- ( ) 8
- ( ) 9
- ( ) 10
- ( ) 11
- ( ) 12

218. What type of cake or cookies have you consumed on such a day? *

Mark only one oval per row.

	Rarely/never	Sometimes	Often	Usually/always
Big cookies (stuffed cookies, stroopwafel cookies, etc.)	( )	( )	( )	( )
Cake	( )	( )	( )	( )

219. How often have you consumed pastries or pie in the last month? *

Mark only one oval.

- Not used
- 1 day in 4 weeks
- 2-3 days in 4 weeks
- 1 day per week
- 2 days per week
- 3 days per week
- 4 days per week
- 5 days per week
- 6 days per week
- 7 days per week

220. How many pieces have you consumed on such a day?

Mark only one oval.

- 1
- 2
- 3
- 4
- 5
- 6
- 7
- 8
- 9
- 10
- 11
- 12

221. How often have you consumed bonbons in the last month? *

Mark only one oval.

- Not used
- 1 day in 4 weeks
- 2-3 days in 4 weeks
- 1 day per week
- 2 days per week
- 3 days per week
- 4 days per week
- 5 days per week
- 6 days per week
- 7 days per week

222. How many pieces have you consumed on such a day?

Mark only one oval.

- 1
- 2
- 3
- 4
- 5
- 6
- 7
- 8
- 9
- 10
- 11
- 12

223. How often have you consumed chocolade in the last month? *

Mark only one oval.

- Not used
- 1 day in 4 weeks
- 2-3 days in 4 weeks
- 1 day per week
- 2 days per week
- 3 days per week
- 4 days per week
- 5 days per week
- 6 days per week
- 7 days per week

224. How many pieces have you consumed on such a day?

Mark only one oval.

- 1
- 2
- 3
- 4
- 5
- 6
- 7
- 8
- 9
- 10
- 11
- 12

225. What type of chocolate have you consumed on such a day? *

Mark only one oval per row.

	Rarely/never	Sometimes	Often	Usually/always
Dark chocolate	◯	◯	◯	◯
Milk chocolate	◯	◯	◯	◯
White chocolate	◯	◯	◯	◯

226. How often have you consumed choco candy or candybars in the last month? *

For example: Mars, Snickers, Twix, Bounty, M&M's, etc.

Mark only one oval.

- ◯ Not used
- ◯ 1 day in 4 weeks
- ◯ 2-3 days in 4 weeks
- ◯ 1 day per week
- ◯ 2 days per week
- ◯ 3 days per week
- ◯ 4 days per week
- ◯ 5 days per week
- ◯ 6 days per week
- ◯ 7 days per week

227. How many pieces/servings have you consumed on such a day?

1 serving = 1 candy bar or 1 serving of choco candy (for example 1 package of M&M's).

Mark only one oval.

- 1
- 2
- 3
- 4
- 5
- 6
- 7
- 8
- 9
- 10
- 11
- 12

228. What size was your candybar usually?

Mark only one oval.

- Miniature (celebrations) or 6 M&M's
- Small candybar or mini package M&M's
- Medium candybar or medium package M&M's
- Large or kingsize candybar or big package M&M's (family package)

229. How often have you consumed chewing gum in the last month? *

Mark only one oval.

- Not used
- 1 day in 4 weeks
- 2-3 days in 4 weeks
- 1 day per week
- 2 days per week
- 3 days per week
- 4 days per week
- 5 days per week
- 6 days per week
- 7 days per week

230. How many pieces have you consumed on such a day?

Mark only one oval.

- 1
- 2
- 3
- 4
- 5
- 6
- 7
- 8
- 9
- 10
- 11
- 12

231. How often have you consumed candy in the last month? *

For example: winegums, licorice, peppermint, etc.

Mark only one oval.

- Not used
- 1 day in 4 weeks
- 2-3 days in 4 weeks
- 1 day per week
- 2 days per week
- 3 days per week
- 4 days per week
- 5 days per week
- 6 days per week
- 7 days per week

232. How many pieces have you consumed on such a day?

Mark only one oval.

- 1-2
- 3-4
- 5-6
- 7-8
- 9-10
- 11-12
- 13-14
- 15-16
- 17-18
- 19-20

233. What type of candy have you consumed on such a day? *

Mark only one oval per row.

	Rarely/never	Sometimes	Often	Usually/always
Licorice	⬭	⬭	⬭	⬭
Other types of candy	⬭	⬭	⬭	⬭

Savory snacks

234. How often have you consumed savory snacks in the last month? *

For example: croquette, sausage pastries, bitterballen, etc.

Mark only one oval.

- Not used
- 1 day in 4 weeks
- 2-3 days in 4 weeks
- 1 day per week
- 2 days per week
- 3 days per week
- 4 days per week
- 5 days per week
- 6 days per week
- 7 days per week

235. How many pieces have you consumed on such a day?

Mark only one oval.

- ⬭ 1
- ⬭ 2
- ⬭ 3
- ⬭ 4
- ⬭ 5
- ⬭ 6
- ⬭ 7
- ⬭ 8
- ⬭ 9
- ⬭ 10
- ⬭ 11
- ⬭ 12

236. What type of savory snacks have you consumed on such a day? *

Mark only one oval per row.

	Rarely/never	Sometimes	Often	Usually/always
Fried savory snacks	⬭	⬭	⬭	⬭
Non-fried savory snacks	⬭	⬭	⬭	⬭

237. How often have you consumed mayonnaise, ketchup or other cold sauces (added to savory snacks) in the last month? *

Mark only one oval.

- Not used
- 1 day in 4 weeks
- 2-3 days in 4 weeks
- 1 day per week
- 2 days per week
- 3 days per week
- 4 days per week
- 5 days per week
- 6 days per week
- 7 days per week

238. How many tablespoons have you consumed on such a day?

Mark only one oval.

- 1
- 2
- 3
- 4
- 5
- 6
- 7
- 8
- 9
- 10
- 11
- 12

239. What type of cold sauces have you consumed on such a day? *

Mark only one oval per row.

	Rarely/never	Sometimes	Often	Usually/always
Mayonnaise	◯	◯	◯	◯
Halvanaise, fries sauce or other non-red sauces	◯	◯	◯	◯
Tomato ketchup or other red sauces	◯	◯	◯	◯

240. How often have you consumed peanuts or nuts (as snack) in the last month? *

Mark only one oval.

- ◯ Not used
- ◯ 1 day in 4 weeks
- ◯ 2-3 days in 4 weeks
- ◯ 1 day per week
- ◯ 2 days per week
- ◯ 3 days per week
- ◯ 4 days per week
- ◯ 5 days per week
- ◯ 6 days per week
- ◯ 7 days per week

241. How many servings have you consumed on such a day?

Mark only one oval.

- 1
- 2
- 3
- 4
- 5
- 6
- 7
- 8
- 9
- 10
- 11
- 12

242. What type of peanuts or nuts have you consumed on such a day? *

Mark only one oval per row.

	Rarely/never	Sometimes	Often	Usually/always
Peanuts or bar mix				
Nuts, nut mix, trail nuts				

243. How often have you consumed crisps or pretzels in the last month? *

Mark only one oval.

- Not used
- 1 day in 4 weeks
- 2-3 days in 4 weeks
- 1 day per week
- 2 days per week
- 3 days per week
- 4 days per week
- 5 days per week
- 6 days per week
- 7 days per week

244. How many servings have you consumed on such a day?

Mark only one oval.

- 1
- 2
- 3
- 4
- 5
- 6
- 7
- 8
- 9
- 10
- 11
- 12

245. How often have you consumed cheese (as snack) in the last month? *

For example: cheese cubes or cheese on crackers.

Mark only one oval.

- Not used
- 1 day in 4 weeks
- 2-3 days in 4 weeks
- 1 day per week
- 2 days per week
- 3 days per week
- 4 days per week
- 5 days per week
- 6 days per week
- 7 days per week

246. How many cheese cubes have you consumed on such a day?

Mark only one oval.

- 1
- 2
- 3
- 4
- 5
- 6
- 7
- 8
- 9
- 10
- 11
- 12

247. How often have you consumed cold cuts (as snack) in the last month? *

For example: cold cuts on crackers.

Mark only one oval.

- Not used
- 1 day in 4 weeks
- 2-3 days in 4 weeks
- 1 day per week
- 2 days per week
- 3 days per week
- 4 days per week
- 5 days per week
- 6 days per week
- 7 days per week

248. How many slices have you consumed on such a day?

Mark only one oval.

- 1
- 2
- 3
- 4
- 5
- 6
- 7
- 8
- 9
- 10
- 11
- 12

249. What type of cold cuts have you consumed on such a day? *

Mark only one oval per row.

	Rarely/never	Sometimes	Often	Usually/always
Filet americain				
Boiled liver				
Sausage: liver sausage, pate, liver pate, liver cheese				
Ham: raw, smoked, boiled, processed				
Roast beef, chicken breast				
Sausage: cervelate, salami, etc.				
Other types of culd cuts				

250. How often have you consumed salad spreads on crackers in the last month? *

Mark only one oval.

- Not used
- 1 day in 4 weeks
- 2-3 days in 4 weeks
- 1 day per week
- 2 days per week
- 3 days per week
- 4 days per week
- 5 days per week
- 6 days per week
- 7 days per week

251. How many crackers with spread have you consumed on such a day?

Mark only one oval.

- 1
- 2
- 3
- 4
- 5
- 6
- 7
- 8
- 9
- 10
- 11
- 12

Juices, soda & water

252. How often have you consumed fruit or vegetable juice in the last month? *

Mark only one oval.

- Not used
- 1 day in 4 weeks
- 2-3 days in 4 weeks
- 1 day per week
- 2 days per week
- 3 days per week
- 4 days per week
- 5 days per week
- 6 days per week
- 7 days per week

253. How many glasses have you consumed on such a day?

Mark only one oval.

- 1
- 2
- 3
- 4
- 5
- 6
- 7
- 8
- 9
- 10
- 11
- 12

254. What type of juices have you consumed on such a day? *

Mark only one oval per row.

	Rarely/never	Sometimes	Often	Usually/always
Fruit juice (fresh or prepackaged)				
Fruit juice, duo fruit, multivitamin juice				
Vegetable juice				

255. How often have you consumed water in the last month? *

Mark only one oval.

- Not used
- 1 day in 4 weeks
- 2-3 days in 4 weeks
- 1 day per week
- 2 days per week
- 3 days per week
- 4 days per week
- 5 days per week
- 6 days per week
- 7 days per week

256. How many glasses have you consumed on such a day?

Mark only one oval.

- 1
- 2
- 3
- 4
- 5
- 6
- 7
- 8
- 9
- 10
- 11
- 12

257. What type of water have you consumed on such a day? *

Mark only one oval per row.

	Rarely/never	Sometimes	Often	Usually/always
Tap water	◯	◯	◯	◯
Mineral or bottled water	◯	◯	◯	◯

258. How often have you consumed light soda or lemonade in the last month? *

Mark only one oval.

- ◯ Not used
- ◯ 1 day in 4 weeks
- ◯ 2-3 days in 4 weeks
- ◯ 1 day per week
- ◯ 2 days per week
- ◯ 3 days per week
- ◯ 4 days per week
- ◯ 5 days per week
- ◯ 6 days per week
- ◯ 7 days per week

259. How many glasses have you consumed on such a day?

Mark only one oval.

- 1
- 2
- 3
- 4
- 5
- 6
- 7
- 8
- 9
- 10
- 11
- 12

260. What type of light soda have you consumed on such a day? *

Mark only one oval per row.

	Rarely/never	Sometimes	Often	Usually/always
Light soda or carbonated lemonade				
Light soda or non-carbonated lemonade				

261. How often have you consumed soda or lemonade in the last month? *

Mark only one oval.

- Not used
- 1 day in 4 weeks
- 2-3 days in 4 weeks
- 1 day per week
- 2 days per week
- 3 days per week
- 4 days per week
- 5 days per week
- 6 days per week
- 7 days per week

262. How many glasses have you consumed on such a day?

Mark only one oval.

- 1
- 2
- 3
- 4
- 5
- 6
- 7
- 8
- 9
- 10
- 11
- 12

263. What type of soda have you consumed on such a day? *

Mark only one oval per row.

	Rarely/never	Sometimes	Often	Usually/always
Soda or carbonated lemonade	◯	◯	◯	◯
Soda or non-carbonated lemonade	◯	◯	◯	◯

264. How often have you consumed energy drinks in the last month? *

Mark only one oval.

- ◯ Not used
- ◯ 1 day in 4 weeks
- ◯ 2-3 days in 4 weeks
- ◯ 1 day per week
- ◯ 2 days per week
- ◯ 3 days per week
- ◯ 4 days per week
- ◯ 5 days per week
- ◯ 6 days per week
- ◯ 7 days per week

265. How many glasses have you consumed on such a day?

Mark only one oval.

- 1
- 2
- 3
- 4
- 5
- 6
- 7
- 8
- 9
- 10
- 11
- 12

Alcoholic beverages

266. How often have you consumed beer in the last month? *

For example: pilsner, double, tripple, etc.

Mark only one oval.

- Not used
- 1 day in 4 weeks
- 2-3 days in 4 weeks
- 1 day per week
- 2 days per week
- 3 days per week
- 4 days per week
- 5 days per week
- 6 days per week
- 7 days per week

267. How many glasses have you consumed on such a day?

1 bottle = 1.5 glass.

Mark only one oval.

- 1-2
- 3-4
- 5-6
- 7-8
- 9-10
- 11-12
- 13-14
- 15-16
- 17-18
- 19-20

268. How often have you consumed non-alcoholic beer in the last month? *

Mark only one oval.

- Not used
- 1 day in 4 weeks
- 2-3 days in 4 weeks
- 1 day per week
- 2 days per week
- 3 days per week
- 4 days per week
- 5 days per week
- 6 days per week
- 7 days per week

269. How many glasses have you consumed on such a day?

1 bottle = 1.5 glass.

Mark only one oval.

- 1-2
- 3-4
- 5-6
- 7-8
- 9-10
- 11-12
- 13-14
- 15-16
- 17-18
- 19-20

270. How often have you consumed mix drinks in the last month? *

For example: breezer, etc.

Mark only one oval.

- Not used
- 1 day in 4 weeks
- 2-3 days in 4 weeks
- 1 day per week
- 2 days per week
- 3 days per week
- 4 days per week
- 5 days per week
- 6 days per week
- 7 days per week

271. How many glasses have you consumed on such a day?

Mark only one oval.

- 1
- 2
- 3
- 4
- 5
- 6
- 7
- 8
- 9
- 10
- 11
- 12

272. How often have you consumed wine in the last month? *

Mark only one oval.

- Not used
- 1 day in 4 weeks
- 2-3 days in 4 weeks
- 1 day per week
- 2 days per week
- 3 days per week
- 4 days per week
- 5 days per week
- 6 days per week
- 7 days per week

273. How many glasses have you consumed on such a day?

Mark only one oval.

- 1
- 2
- 3
- 4
- 5
- 6
- 7
- 8
- 9
- 10
- 11
- 12

274. How often have you consumed fortified wine in the last month? *

For example: sherry, port, vermouth, etc.

Mark only one oval.

- Not used
- 1 day in 4 weeks
- 2-3 days in 4 weeks
- 1 day per week
- 2 days per week
- 3 days per week
- 4 days per week
- 5 days per week
- 6 days per week
- 7 days per week

275. How many glasses have you consumed on such a day?

Mark only one oval.

- 1
- 2
- 3
- 4
- 5
- 6
- 7
- 8
- 9
- 10
- 11
- 12

276. How often have you consumed spirits in the last month? *

Mark only one oval.

- Not used
- 1 day in 4 weeks
- 2-3 days in 4 weeks
- 1 day per week
- 2 days per week
- 3 days per week
- 4 days per week
- 5 days per week
- 6 days per week
- 7 days per week

277. How many glasses have you consumed on such a day?

Mark only one oval.

- 1
- 2
- 3
- 4
- 5
- 6
- 7
- 8
- 9
- 10
- 11
- 12

Additional questions

278. How often have you consumed herbs and spices in the last month? *

Mark only one oval.

- Not used
- 1 day in 4 weeks
- 2-3 days in 4 weeks
- 1 day per week
- 2 days per week
- 3 days per week
- 4 days per week
- 5 days per week
- 6 days per week
- 7 days per week

279. How often have you consumed bouillon cubes/powder in the last month? *

Mark only one oval.

- Not used
- 1 day in 4 weeks
- 2-3 days in 4 weeks
- 1 day per week
- 2 days per week
- 3 days per week
- 4 days per week
- 5 days per week
- 6 days per week
- 7 days per week

280. How often have you consumed soy sauce in the last month? *

Mark only one oval.

- Not used
- 1 day in 4 weeks
- 2-3 days in 4 weeks
- 1 day per week
- 2 days per week
- 3 days per week
- 4 days per week
- 5 days per week
- 6 days per week
- 7 days per week

281. How often have you consumed superfoods in the last month? *

Mark only one oval.

- Not used
- 1 day in 4 weeks
- 2-3 days in 4 weeks
- 1 day per week
- 2 days per week
- 3 days per week
- 4 days per week
- 5 days per week
- 6 days per week
- 7 days per week

282. How often have you consumed fast food / take out in the last month? *

Mark only one oval.

- Not used
- 1 day in 4 weeks
- 2-3 days in 4 weeks
- 1 day per week
- 2 days per week
- 3 days per week
- 4 days per week
- 5 days per week
- 6 days per week
- 7 days per week

283. How often have you consumed mildly spicy foods in the last month? *

Mark only one oval.

- Not used
- 1 day in 4 weeks
- 2-3 days in 4 weeks
- 1 day per week
- 2 days per week
- 3 days per week
- 4 days per week
- 5 days per week
- 6 days per week
- 7 days per week

284. How often have you consumed very spicy foods in the last month? *

Mark only one oval.

- Not used
- 1 day in 4 weeks
- 2-3 days in 4 weeks
- 1 day per week
- 2 days per week
- 3 days per week
- 4 days per week
- 5 days per week
- 6 days per week
- 7 days per week

Thank you!

Dear participant, thank you for filling out this questionnaire. If you have any comments on the GINQ-FFQ, please mention it in the comment box below.

285. Comment box:

This content is neither created nor endorsed by Google.

Google Forms
