# Supplementary material for: Can Diet Quality Be Associated with Disease Activity in a Prospective Dutch Inflammatory Bowel Disease Cohort?
Source: Nutrients. 2025 Apr 8;17(8):1298. doi: 10.3390/nu17081298 (PMC12029865; doi:10.3390/nu17081298)
Supplement: Supplementary file 1 [file nutrients-17-01298-s001.zip › nutrients-3549635-supplementary/Supplementary tables Nutrients.pdf]

Supplemental material

Can diet quality be associated with disease activity in a prospective Dutch IBD cohort?

Supplement Table S1: Food items and food groups derived from the GINQ-FFQ:

Food group		Food Items	
Dutch	English	Dutch	English
Melk en melkproducten	Dairy and dairy products (g)	Drinkontbijt (Goede Morgen Vifit)	Breakfast drink (Goede Morgen Vifit)
		Melk volle gecondenseerd met suiker (blik)	Milk full fat condensed with sugar (tinned)
		Melk volle, halfvolle en magere	Milk whole, semi-skimmed and skimmed
		Karnemelk	Buttermilk
		Lactose vrije melk (halfvol)	Lactose free milk (semi-skimmed)
		Chocolademelk (volle) en met zoetstof (Optimel)	Chocolate flavoured milk (full fat) and with sweetener (Optimel)
		Yoghurtdrank (Fristi), met zoetstof (Topvit) en naturel (Actimel)	Yoghurt drink (Fristi), with sweetener (Topvit) and plain (Actimel)
		Milkshake met vers fruit	Milkshake with fresh fruit
		Volle Vla (overige smaken)	Custard full fat (several flavours)
		Volle, halfvolle en magere yoghurt met vruchten	Full fat, half fat and low fat yoghurt with fruit
		Havermoutpap bereid met volle melk, ongezoet	Porridge prepared w whole milk, unsweetened
		Roomijs (vanille-)	Ice cream with dairy
		Slagroom onbereid en met suiker geklopt	Whipped Cream unprepared and whipped with added sugar
		Koffiemelk volle en halfvolle	Coffee creamer full fat and half fat
		Room slag- onbereid	Cream whipping unprepared
Kaas	Cheese (g)	Smeerkaas 40+	Cheese spread 40+
		Roomkaas (zachte Paturain)	Cream cheese cream (soft Paturain)
		Kaas 20+, 48 +	Cheese 20+, 48+
Eieren	Eggs (g)	Kippenei, rauw	Chicken egg, raw
Graanproducten en bindmiddelen	Grain products and binding agents (g)	Muesli met vruchten	Muesli with fruit
		Muesli naturel	Muesli plain
		Tarwezemelen	Wheat bran
		Griesmeel	Semolina

		Ontbijtproduct Cornflakes (Kellogg's)	Breakfast cereal Cornflakes (Kellogg's)
		Ontbijtproduct All-Bran flakes (Kellogg's)	Breakfast cereal All-Bran flakes (Kellogg's)
		Ontbijtproduct Brinta	Breakfast cereal Brinta
		Pannenkoek bereid met margarine	Pancake prep with margarin
		Pasta witte en volkoren	Pasta white and wholemeal
		Rijst witte en zilvervlies	Rice white and brown
		Couscous gekookt	Couscous boiled
Brood	Bread (g)	Beschuit naturel	Crisp bakes Dutch white
		Croissant	Croissant
		Zacht broodje wit, tarwe en volkoren	Soft roll white, brown and whole grain
		Hard broodje meergranen	Hard roll multigrain
		Krentenbol	Bun currant/raisin
		Brood wit, tarwe, volkoren, meergranen en rogge (licht en donker)	Bread white, brown, whole grain, multigrain and rye (light and dark)
Gebak en koek	Pastries and cookies (g)	Ontbijtkoek	Dutch spiced cake
		Bastognekoek	Shortbread biscuit shortbread (Bastogne)
		Voedingsbiscuit (Liga Evergreen krenten)	Fortified biscuit with currants (Liga Evergreen)
		Stroopwafel	Syrup waffle
		Cake zonder roomboter	Cake without butter
		Slagroomtaart	Gateau with whipped cream
Hartig broodbeleg	Savory sandwich spread (g)	Pindakaas	Peanut butter
		Sandwich spread naturel	Sandwich spread original
		Kip-kerrie salade	Chicken curry salad
Hartige sauzen	Savory sauces (g)	Slasaus 25% olie	Salad cream 25% oil
		Dressing naturel zonder olie	Salad dressing natural without oil
		Satésaus, kant-en-klaar	Peanut sauce, ready-made
		Tomatensaus, kant-en-klaar	Tomato sauce tomato, ready-made
		Jus 25% vet, gebonden bereid met juspoeder	Gravy 25% fat, thickened prepared with instant powder
		Mayonaise	Mayonnaise
		Halvanaise	Mayonnaise low fat 40% oil
		Tomatenketchup	Tomato ketchup

Hartige snacks en zoutjes	Savory snacks (g)	Vleeskroket, bereid met vloeibaar frituurvet	Meat ragout croquette, deep-fried in liquid fat
		Worstenbroodje	Sausage roll with bread dough pastry
		Chips	Crisps potato
Suiker, snoep, zoet beleg en zoete sauzen	Sugar. confectionery. sweet sauces and sweet spreads (g)	Chocoladehagelslag	Chocolate confetti
		Melkchocoladepasta	Chocolate milk spread
		Zoet broodbeleg	Sweet spread
		Kristalsuiker	Granulated sugar
		Honing	Honey
		Bonbon	Chocolates filled/Belgium chocolate
		Chocolade puur, melk en wit	Chocolate dark, milk and white
		Candybar (Mars)	Candy bar (Mars)
		Kauwgom	Chewing gum
		Drop	Liquorice Dutch type
		Winegum/fruitgom	Wine gum/ fruit gum
Fruit	Fruit (g)	Mandarijn	Mandarin
		Sinaasappel	Orange
		Grapefruit	Grapefruit
		Citrus fruit (overig)	Citrus fruit (other)
		Aardbeien	Strawberries
		Appel met schil	Apple with skin
		Banaan	Banana
		Rode bessen	Redcurrants
		Druiven met schil	Grapes w skin
		Kersen	Cherries
		Groene kiwi	Green kiwi
		Netmeloen	Netted melon
		Peer met schil	Pear with skin
		Fruitsalade	Fruit
		Abrikozen, gedroogd	Apricots, dried
Noten en zaden	Nuts and seeds (g)	Pinda's, gezouten en ongezouten	Peanuts, salted and unsalted
		Gemengde noten, ongezouten	Mixed nuts, unsalted

		Lijnzaad	Flax seeds
		Zonnebloempitten	Sunflower seeds
Groenten	Vegetables (g)	Soepgroenten	Vegetables for soup
		Ui, rauw en gebakken	Onions, raw and fried in vegetable oil
		Andijvie	Endive
		Biet rode	Beetroot
		Bloemkool	Cauliflower
		Broccoli	Broccoli
		Champignon	Mushroom
		Rode paprika, rauw en gekookt	Red bell pepper raw and boiled
		Prei	Leek
		Rode, witte en spits- kool	Red, white and oxheart cabbage
		Sperziebonen	Haricots verts
		Spinazie	Spinach
		Tomaat, rauw en gekookt	Tomato, raw and boiled
		Witlof	Chicory
		Wortel, rauw en gekookt	Carrot, raw and boiled
		Hollandse roerbakgroente	Dutch vegetables for stir fry
		Kropsla	Butterhead lettuce
		Komkommer zonder schil	Cucumber without skin
Peulvruchten	Legumes (g)	Bonen bruine blik	Beans brown canned
Aardappelen en knolgewassen	Potatoes and root vegetables (g)	Aardappelen zonder schil	Potatoes without skin
		Frites bereid	Chips prepared
		Aardappelschijfjes, diepvries gefrituurd in plantaardige olie	Sliced potatoes, frozen deep-fried in vegetable oil
Samengestelde gerechten	Ready-made meals (g)	Pizza met kaas en groenten, diepvries	Pizza with cheese and vegetables, frozen
		Stamppot wortel/ui zonder vlees	Hotchpotch carrots/onions without meat
		Lasagne Bolognese, koelverse maaltijd	Lasagna Bolognese, ready to eat
		Nasi goreng met ei	Fried rice Nasi goreng with egg
		Groenteschotel met vlees turlu, Turks bereid	Vegetable dish with meat turlu, Turkish prepared
Soepen	Soups (g)	Maaltijdsoep met peulvruchten zonder vlees	Main course with legumes without meat
Kruiden en specerijen	Herbs and spices (g)	Mosterd	Mustard

		Sambal Oelek	Pepper red hot paste
Vetten en oliën	Fats and oils (g)	Margarine 80% vet waarvan < 24g verzadigd, ongezouten en gezouten	Margarine 80% fat < 24g saturated, unsalted and salted
		Bak- en braadvet vloeibaar 97% vet waarvan <17g verzadigd, ongezouten en gezouten	Cooking fat liquid 97% fat <17g saturated, unsalted and salted
		Boter ongezouten	Butter, unsalted
		Halfvolle boterproduct	Half-fat butter product
		Halvarineproduct Blue band goede start, Gouda's Glorie Lichtste Pondje, Becel Proactief, 35% vet waarvan <10g verzadigd	Low fat margarine Blue band goede start, Gouda's Glorie Lichtste Pondje, Becel proactief, 35% fat <10g saturated
		Olijfolie	Olive oil
		Kokosolie	Coconut oil
		Plantaardige olie	Vegetable oil
Vis	Fish (g)	Lekkerbekje, gefrituurd in plantaardige olie	White fish fillet in batter, deep-fried in vegetable oil
		Vissticks	Fish fingers
		Vis, 0-5g en >5g vet	Fish, 0-5g and >5g fat
		Zalm, gerookt	Salmon, smoked
		Haring, gezouten	Herring, salted
		Hollandse Garnalen	Dutch shrimps
		Mosselen	Mussels
		Jacobsschelpen	Coquilles St. Jacques
Vlees en gevogelte	Meat and poultry (g)	Rundvlees, <5g, >5g en >10g vet	Beef <5g, >5g and > 10g fat
		Varkensvlees <5g, 5-14g en >19g vet	Pork <5g, 5-14g and >19g fat
		Varkensschnitzel, gepaneerd	Pork schnitzel, breaded
		Kipfilet	Chicken fillet
		Kipschnitzel, gepaneerd	Chicken schnitzel, breaded
		Half om half gehakt	Minced beef/pork
		Hamburger	Hamburger
		Varkenslever	Pork liver pork
		Schapenvlees <10g vet	Mutton <10g fat

Vleesvervangers en zuivelvervangers	Meat and dairy substitutes (g)	Sojadrink, naturel en diverse smaken (Alpro)	Soy drink, natural and several flavours (Alpro)
		Sojadessert (Alpro)	Soy dessert (Alpro)
Vleeswaren	Cold cuts (g)	Filet americain	Spiced beef steak tartare
		Gekookte varkenslever (vleeswaar)	Boiled pork liver (processed meat product)
		Leverworst	Liver sausage
		Vleeswaren <10g vet	Processed meat products <10 g fat
		Kipfilet (vleeswaar)	Chicken (processed meat product)
		Worst (exclusief leverproducten)	Sausage (liver products excluded)
		Vleeswaren 10-20g vet (exclusief leverproducten)	Processed meat prod 10-20g fat (liver products excluded)
		Gekookte worst gekookte	Cooked sausage
Alcoholische dranken	Alcoholic beverages (g)	Bier	Beer
		Breezer	Breezer
		Rode wijn	Red wine red
		Sherry	Sherry
		Jonge jenever	Dutch young gin
Niet-alcoholische dranken	Non-alcoholic beverages	Koffie en oploskoffie	Coffee and instant powder coffee
		Thee	Tea
		Sinaasappelsap van concentraat	Orange juice from concentrate
		Vruchtendrank, 2 of meer vruchten	Fruit juice, minimal 2 fruits
		Tomaten-groentesap	Tomato/vegetable juice
		Water met 50-100mg calcium per liter	Water with 50-100mg calcium per litre
		Mineraalwater, met en zonder koolzuur	Mineral water, sparkling and not sparkling
		Vruchtenlimonade, light en dubbelfris	Fruit lemonade, light and Dubbelfris
		Rivella	Soft drink with milk serum and sweetener (Rivella)
		Frisdrank zonder cafeïne	Soft drink without caffeine
		Energydrink (Red Bull)	Energy drink (Red Bull)
		Alcoholvrij bier <0,1 vol%	Alcohol free beer <0,1 vol%
Diversen	Miscellaneous (g)	Gistextract Marmite	Yeast extract Marmite

Supplement Table S2. Disease specific characteristics

	IBD Total n=191	Disease activity		p-value
		**Remission n=138**	**Active disease [a] n=53**	
Age at diagnosis (n, %)				0.289
< 17 y	22 (11.5)	19 (13.8)	3 (5.7)	
17-40 y	131 (68.6)	92 (66.7)	39 (73.6)	
>40 y	38 (19.9)	27 (19.6)	11 (20.8)	
Crohn's Disease (n, %)				
Disease location				0.363
Terminal ileum	30 (29.1)	24 (32.4)	6 (20.7)	
Colon	24 (23.3)	15 (20.3)	9 (31.0)	
Ileocolonic	49 (47.6)	35 (48.3)	14 (48.3)	
Upper GI-involvement[b]	7 (6.8)	4 (5.4)	3 (10.3)	0.370
Perianal disease[c]	35 (34.0)	28 (37.8)	7 (24.1)	0.187
Behaviour				0.212
Inflammatory	49 (47.6)	35 (47.3)	14 (48.3)	
Structuring	33 (32.0)	21 (28.4)	12 (41.1)	
Penetrating	21 (20.4)	18 (24.3)	3 (10.3)	
Ulcerative Colitis (n, %)				
Disease extension				0.214
Proctitis	7 (8.0)	7 (10.9)	0 (0)	
Left-sided colitis	37 (42.0)	27 (42.2)	10 (41.7)	
Pancolitis	44 (50.0)	30 (46.9)	14 (58.3)	
Medication use				
No medication	18 (9.4)	17 (12.3)	1 (1.9)	**0.027***
(local) corticosteroids	9 (4.7)	3 (2.2)	6 (11.3)	**0.008***
Amino salicylates[d]	42 (22.0)	36 (26.1)	6 (11.3)	**0.027***
Thiopurines / MTX[d]	31 (16.2)	26 (18.8)	5 (9.4)	0.114
Biologicals/Small molecules	106 (55.5)	65 (47.1)	41 (77.4)	**<0.001***

Data was presented as absolute numbers of available data (=n) with corresponding percentages. Chi-square (X^2) test was used to compare categorical data. IBD= inflammatory bowel disease. [a]: defined as CRP ≥5 mg/L and/or FCP ≥ 150 µg/g. [b]: Upper GI-involvement (Montreal L4) is presented as a modifier which includes isolated upper GI-involvement and upper GI-involvement in addition to other disease locations. [c]: Perianal disease (Montreal p) is presented as a modifier which includes perianal disease in combination with penetrating disease behaviour and solely perianal disease involvement separate from penetrating disease behaviour. [d]: without biologicals/small molecule usage. *p-value <0.05.

Supplement Table S3. Patient characteristics of 81 participants with dietary intake data at baseline and 1-year follow-up

	IBD Total n=81	Disease activity: Remission n=59	Disease activity: Active disease [a] n=22	*p-value*
Age (years)	52.22 ± 1.726	52.32 ± 1.953	51.95 ± 3.5677	0.925
Female sex (n, %)	45 (55.6)	31 (52.5)	14 (63.6)	0.371
Disease duration (years)	19.00 [8.50 – 29.00]	22.00 [9.50 – 30.50]	13.50 [6.00 – 23.00]	0.140
Age at diagnosis (n, %)				0.342
< 17 y	8 (9.9)	6 (10.2)	2 (9.1)	
17-40 y	56 (69.1)	43 (72.9)	13 (59.1)	
>40 y	17 (21.0)	10 (16.9)	7 (31.8)	
IBD type (CD) (n, %)	47 (58.0)	31 (52.5)	16 (72.7)	0.102
C-reactive protein	2.35 [1.00 – 6.20]	1.60 [1.00 – 3.100]	7.00 [3.50 – 20.30]	**<0.001***
Faecal calprotectin	90.00 [15.00 – 224.00]	28.50 [12.00 – 113.00]	433.00 [167.00 – 1372.00]	**<0.001***
Crohn's Disease (n, %)				
Disease location				0.554
Terminal ileum	12 (25.5)	8 (25.8)	4 (25.0)	
Colon	16 (34.0)	9 (29.0)	7 (43.8)	
Ileocolonic	19 (40.4)	14 (45.2)	5 (31.3)	
Upper GI-involvement [b]	3 (6.4)	1 (3.2)	2 (12.5)	0.218
Perianal disease [c]	15 (31.9)	13 (41.9)	2 (12.5)	**0.040***
Behaviour				0.183
inflammatory	21 (44.7)	14 (45.2)	7 (43.8)	
Structuring	17 (36.2)	9 (29.0)	8 (50.0)	
Penetrating	9 (19.1)	8 (25.8)	1 (6.3)	
Ulcerative Colitis (n, %)				
Disease extension				0.567
Proctitis	3 (8.8)	3 (10.7)	0 (0.0)	
Left-sided colitis	12 (35.3)	9 (32.1)	3 (50.0)	
Pancolitis	19 (55.9)	16 (57.1)	3 (50.0)	
Medication use				
No medication	10 (12.3)	9 (15.3)	1 (4.5)	0.193
(local) corticosteroids	2 (2.5)	0 (0.0)	2 (9.1)	**0.019***
Amino salicylates [d]	17 (21.0)	16 (27.1)	1 (4.5)	**0.026***
Thiopurines [d]	10 (12.3)	10 (16.9)	0 (0.0)	**0.039***
Biologicals/Small molecules	45 (55.6)	27 (45.8)	18 (81.8)	**0.004***

Data was presented as mean ± standard deviation for normally distributed data, median [interquartile ranges] for skewed data and absolute numbers (%). Independent t-test, Mann-Whitney U test or Chi-square (X^2) test was used to compare between active disease and remission. *p-value <0.05. IBD= Inflammatory Bowel Disease. n= number. [a]: defined as CRP ≥5 mg/L and/or FCP ≥ 150 μg/g. [b]: Upper GI-involvement (Montreal L4) is presented as a modifier which includes isolated upper GI-involvement and upper GI-involvement in addition to other disease locations. [c]: Perianal disease (Montreal p) is presented as a modifier which includes perianal disease in combination with penetrating disease behavior and solely perianal disease involvement separate from penetrating disease behavior. [d]: without biologicals/small molecule usage.

Supplement Table S4. Nutrient intake baseline versus follow-up (n=81)

	Baseline (n=81)	1 year (n=81)	p-value
Macronutrients			
Energy total (kcal)	1961.56 ± 541.70	2066.59 ± 571.25	0.059
Protein			
- Total (g)	75.17 [64.09 - 90.68]	80.81 [62.32 - 103.42]	0.401
- Plant (g)	25.38 [19.43 - 33.73]	26.63 [21.20 - 38.30]	0.079
- Animal (g)	52.58 ± 23.11	53.57 ± 25.62	0.628
Carbohydrates			
- Total (g)	175.17 [132.33 - 218.20]	178.61 [137.34 - 227.26]	0.278
- Sugar (g)	80.48 ± 26.94	83.64 ± 32.60	0.336
- Fibre (g)	22.05 [16.57 - 27.48]	23.01 [17.00 - 28.88]	0.058
Alcohol			
- (g)	1.75 [0.00 - 6.66]	1.66 [0.00 - 6.70]	0.791
Water (g)	1841.08 [1609.10 - 2177.03]	1864.49 [1491.62 - 2200.12]	0.816
Fat			
- Total (g)	90.45 [76.33 - 103.63]	93.57 [76.85 - 120.24]	0.086
- Total (E%)	42.51 ± 6.33	43.91 ± 5.64	**0.045***
- Saturated fat (g)	30.19 ± 13.56	30.53 ± 13.54	0.750
- MUFA (g)	36.23 [30.47 - 42.67]	40.62 [33.55 - 50.77]	**0.008***
- PUFA (g)	17.66 ± 7.42	19.74 ± 8.23	**0.024***
- PUFA N3 (g)	2.72 [2.06 - 3.98]	2.97 [2.28 - 4.27]	0.170
- PUFA N6 (g)	12.72 [9.70 - 15.85]	13.49 [10.99 - 16.68]	0.100
- Trans fatty acids	0.56 [0.41 - 0.83]	0.56 [0.39 - 0.79]	0.794
- Cholesterol	214.47 [173.22 - 297.22]	214.46 [158.22 - 287.10]	0.923
Micronutrients			
Sodium (mg)	1774.60 ± 645.44	1773.52 ± 632.91	0.984
Potassium (mg)	3021.70 ± 793.56	3246.14 ± 994.68	**0.014***
Calcium (mg)	859.97 [698.66 - 1091.10]	845.36 [608.94 - 1179.08]	0.968
Phosphate (mg)	1459.15 ± 470.00	1541 ± 551.80	0.111
Magnesium	320.12 [263.93 - 365.60]	315.44 [260.70 - 411.84]	0.168
Iron			
- Total (mg)	9.59 ± 2.98	10.00 ± 3.07	0.183
- Heme (mg)	0.98 ± 0.62	0.93 ± 0.65	0.361
- Non-heme (mg)	8.60 ± 2.83	9.07 ± 2.99	0.107
Zinc (mg)	9.72 [8.65 - 11.27]	10.22 [7.37 - 12.67]	0.660
Vitamins			
- A (RAE) (mug)	780.13 [648.17 - 1034.32]	893.82 [700.32 - 1108.06]	0.125#
- D (mug)	3.57 [2.08 - 4.45]	3.32 [2.18 - 4.69]	0.979#
- E (mug)	13.25 [10.20 - 16.13]	14.44 [11.64 - 17.21]	**0.041***
- B1 (mg)	0.93 ± 0.34	1.02 ± 0.39	**0.012***
- B2 (mg)	1.15 [0.93 - 1.57]	1.24 [0.87 - 1.62]	0.235

-	B3 (mg)	19.23 [16.50 - 24.71]	21.21 [16.69 - 26.89]	0.233
-	B6 (mg)	1.60 ± 0.48	1.67 ± 0.53	0.187
-	B11 (mug)	238.94 [204.25-279.56]	235.37 [196.25-288.34]	0.175
-	B12 (mug)	5.11 ± 2.50	4.88 ± 2.48	0.316
-	C (mg)	74.58 [50.89 - 101.94]	71.6 [51.67 - 103.94]	0.395

Values are shown as Median [IQR] or Mean ± SD. IQR= interquartile range, SD= standard deviation. Paired t-test or Wilcoxon signed-rank test was used to compare between active disease and remission. *p-value <0.05. n= number. E%=Energy percent= ((nutrient*Atwater factor)/Energy total)*100. MUFA= Monounsaturated fatty acids, PUFA= Polyunsaturated fatty acids, RAE= Retinol Equivalents.

Supplement Table S5. Food group intake baseline versus follow-up (n=81)

	**Baseline (n=81)**	**1 year (n=81)**	**p-value**
Dairy and dairy products (g)	182.63 [87.75-347.04]	212.98 [97.98-427.00]	0.061
Cheese (g)	27.86 [11.43-42.86]	20.350 [11.79-41.61]	**0.031***
Eggs (g)	14.29 [7.14-28.57]	14.29 [7.14-25.00]	0.595
Grain products and binding agents (g)	53.58 [31.16-91.71]	51.92 [31.98-96.52]	0.544
Bread (g)	66.35 [32.96-130.95]	75.52 [37.05-131.43]	0.245
Pastries and cookies (g)	15.00 [7.14-31.43]	15.98 [8.57-30.54]	0.903
Savory sandwich spread (g)	0.54 [0.00-4.82]	2.14 [0.00-6.96]	0.101
Savory sauces (g)	7.77 [3.57-13.04]	7.41 [4.29-14.11]	0.767
Savory snacks (g)	10.36 [5.71-20.00]	8.57 [5.18-15.71]	0.426
Sugar. confectionery. sweet sauces and sweet spreads (g)	14.57 [4.54-21.64]	12.50 [4.32-21.51]	0.286
Fruit (g)	150.00 [77.68-258.39]	150.00 [85.71-300.00]	0.982
Nuts and seeds (g)	14.29 [1.79-30.80]	17.86 [4.02-35.71]	0.093
Vegetables (g)	120.09 [74.29-203.04]	137.86 [90.00-228.57]	0.114
Legumes (g)	8.93 [0.00-17.86]	8.93 [0.00-28.57]	0.062
Potatoes and root vegetables (g)	42.86 [26.79-80.36]	55.36 [23.21-75.89]	0.450
Ready-made meals (g)	21.43 [0.00-37.50]	21.43 [0.00-35.71]	0.901
Soups (g)	8.93 [2.23-28.57]	8.93 [2.23-20.41]	0.630
Herbs and spices (g)	0.43 [0.00-1.00]	0.36 [0.00-0.89]	0.997
Fats and oils (g)	31.80 [25.64-39.46]	33.96 [30.00-43.32]	**0.001***
Fish (g)	22.32 [8.93-46.43]	26.61 [8.93-40.00]	0.164
Meat and poultry (g)	97.13 ± 67.85	100.66 ± 69.31	0.538
Meat and dairy substitutes (g)	0.00 [0.00-0.00]	0.00 [0.00-0.00]	0.889
Cold cuts (g)	7.50 [1.07-17.68]	8.57 [0.00-19.29]	0.752
Alcoholic beverages (g)	20.54 [0.00-81.43]	17.86 [0.00-85.71]	0.408
Non-alcoholic beverages (g)	1096.38 ± 438.84	1015.87 ± 420.61	0.091
Miscellaneous (g)	0.00 [0.00-0.00]	0.00 [0.00-0.00]	0.180

Values are shown as Median [IQR] or Mean ± SD. IQR= interquartile range, SD= standard deviation. Paired t-test or Wilcoxon signed-rank test was used to compare between baseline and 1 year. *p-value <0.05. n= number.

Supplement Table S6. Changes in dietary quality scores over the course of one year

		Baseline n=81	1 year n=81	p-value
DII		-0.209 ± 2.218	0.250 ± 2.512	0.063
	UC	0.187 ± 2.021	1.072 ± 2.287	**0.036***
	CD	-0.496 ± 2.330	-0.345 ± 2.522	0.613
HDI-2015				0.366
	Low	73 (90.1)	69 (85.2)	
	Moderate	7 (8.6)	12 (14.8)	
	High	1(1.2)	0 (0.0)	
UC				0.480
	Low	29 (85.3)	26 (76.5)	
	Moderate	4 (11.8)	8 (23.5)	
	High	1 (2.9)	0 (0.0)	
CD				0.564
	Low	44 (93.6)	43 (91.5)	
	Moderate	3 (6.4)	4 (80.5)	
	High	0 (0.0)	0 (0.0)	
MDS		3 [2 - 4]	3 [2 - 4]	0.501
	UC	3 [2 - 4]	3 [2-4]	0.325
	CD	2 [2 - 3.5]	2 [1 – 4]	0.833
UPF (EN%)		41.38 ± 13.78	40.21 ± 14.17	0.235
	UC	39.14 ± 12.57	37.56 ± 14.36	0.371
	CD	42.00 ± 14.51	42.13 ± 13.88	0.446

Data are presented as mean ± SD for normally distributed variables, median [IQR] for skewed data, or as absolute counts (n) with corresponding percentages (%). Paired t-test, Wilcoxon signed-rank test or McNemar's test was used to compare between baseline and 1 year. *p-value <0.05. CD: Crohn's Disease. UC: Ulcerative Colitis. UPF: Ultra-processed food
